# Human liver stem cells express UGT1A1 and improve phenotype of immunocompromised Crigler Najjar syndrome type I mice

**DOI:** 10.1038/s41598-020-57820-2

**Published:** 2020-01-21

**Authors:** Elvira Smeralda Famulari, Victor Navarro-Tableros, Maria Beatriz Herrera Sanchez, Giulia Bortolussi, Marta Gai, Laura Conti, Lorenzo Silengo, Emanuela Tolosano, Ciro Tetta, Andrés Fernando Muro, Giovanni Camussi, Sharmila Fagoonee, Fiorella Altruda

**Affiliations:** 1 Molecular Biotechnology Center, Department of Molecular Biotechnology and Health Sciences, University of Turin, Turin, Italy; 2 2i3T - Società per la gestione dell’incubatore di imprese e per il trasferimento tecnologico dell’Università degli studi di Torino, Scarl and Molecular Biotechnology Center, Turin, Italy; 3 International Centre for Genetic Engineering and Biotechnology (ICGEB), Trieste, Italy; 4 Unicyte srl, Turin, Italy; 5 Department of Medical Sciences, University of Turin, Turin, Italy; 6 Institute of Biostructure and Bioimaging, CNR c/o Molecular Biotechnology Center, Turin, Italy

**Keywords:** Cell biology, Adult stem cells

## Abstract

Crigler Najjar Syndrome type I (CNSI) is a rare recessive disorder caused by mutations in the *Ugt1a1* gene. There is no permanent cure except for liver transplantation, and current therapies present several shortcomings. Since stem cell-based therapy offers a promising alternative for the treatment of this disorder, we evaluated the therapeutic potential of human liver stem cells (HLSC) in immune-compromised NOD SCID Gamma (NSG)/Ugt1^−/−^ mice, which closely mimic the pathological manifestations in CNSI patients. To assess whether HLSC expressed UGT1A1, decellularised mouse liver scaffolds were repopulated with these cells. After 15 days’ culture *ex vivo*, HLSC differentiated into hepatocyte-like cells showing UGT1A1 expression and activity. For the *in vivo* human cell engraftment and recovery experiments, DiI-labelled HLSC were injected into the liver of 5 days old NSG/Ugt1^−/−^ pups which were analysed at postnatal Day 21. HLSC expressed UGT1A1 *in vivo*, induced a strong decrease in serum unconjugated bilirubin, thus significantly improving phenotype and survival compared to untreated controls. A striking recovery from brain damage was also observed in HLSC-injected mutant mice *versus* controls. Our proof-of-concept study shows that HLSC express UGT1A1 *in vivo* and improve the phenotype and survival of NSG/Ugt1^−/−^ mice, and show promises for the treatment of CNSI.

## Introduction

The Crigler Najjar syndrome type I (CNSI; OMIM number 218800) is a rare monogenic disease (0.6 to 1 per 1 000 000 newborns) caused by deficiency in the only enzyme responsible for bilirubin conjugation in the liver, uridine-diphosphate (UDP)-glucuronosyltransferase (UGT) 1A1^1^. The disease is characterised by severe jaundice (elevated blood levels of bilirubin) since birth and a lifelong risk of bilirubin encephalopathy and death by kernicterus if untreated^2,3^. The current treatment for the disease is intensive phototherapy (PT). However, the clinical management is very difficult since CNSI patients need 10–12 hours/day of PT. In addition, patients respond temporarily to PT, as its effectiveness diminishes with age due to skin thickening and decreased surface/body mass ratio, leading to increased risk of unconjugated bilirubin-induced encephalopathy and permanent brain damage^4^. Liver transplantation can improve the prognosis of inherited metabolic diseases, and is currently the only definitive treatment available for CNSI and other severe liver diseases^5^. However, donor organs are scarce.

As an alternative to liver transplantation, cell-based therapy has great potential in the treatment of metabolic liver diseases including CNSI, for the possibility of achieving substitution of diseased hepatic cells and restore function. The metabolic recovery of the hyperbilirubinemic Gunn rat for short periods has been shown after the transplantation of different cell types. For instance, use of primary hepatocytes or immortalized hepatocytes in irradiated/resected liver and non-treated livers resulted, in some cases, in the long term amelioration of hyperbilirubinemia (up to 12 months). Importantly, human neonatal hepatocyte transplantation also provided long-term (6 months) rescue from unconjugated hyperbilirubinemia in adult Gunn rats^6^. These cells showed better engraftment and repopulation capability after transplantation compared to adult cells. Maerckx *et al*. further demonstrated that differentiated adult progenitor cells caused a decrease in serum bilirubin in the Gunn rat for 27 weeks^7^. Other cell types have also been employed in models of CNSI, and are promising for translation into the clinic. For instance, induced pluripotent stem cells reprogrammed from human skin fibroblasts and induced to differentiate into hepatocyte-like cells *in vitro*, expressed UGT1A1 and constituted 2.5–7.5% of the liver when transplanted in Gunn rats, with a reduction in serum bilirubin levels up to 24 weeks post-transplantation^8^. Recently, mesenchymal stem cell-derived from induced pluripotent stem cells (iMSCs) were transplanted in Gunn rat liver after partial hepatectomy and showed engraftment up to 2 months as well as partial recovery from hyperbilirubinemia^9^. The first infusion of human hepatocytes into the liver of a patient with CNSI through the portal vein revealed that the cells survived for 11 months and partially ameliorated liver metabolic function^10^. Hepatic progenitor cells have also been transplanted into a CNSI infant through hepatic artery, and a 2 months follow-up showed decrease in total bilirubin and an increase in conjugated bilirubin levels^11^. Hepatocytes obtained from other sources of cells still have to be tested for their *in vivo* functionality and efficacy in correcting CNSI-related hyperbilirubinemia.

We have previously reported the isolation and characterisation of a population of human liver stem cells (HLSC)^12^. HLSC have mesenchymal stem cell characteristics with partial commitment to hepatic cells. HLSC have a very high propensity to differentiate into hepatocyte-like cells, as indicated by the expression of functional cytochrome P450, albumin and urea production, and downregulation of α-fetoprotein expression. *In vivo*, HLSC engrafted and contributed to regeneration of the liver parenchyma in severe-combined immunodeficient (SCID) mice^12^. Moreover, HLSC differentiated into mature hepatocytes *in vivo* and offered protection from death in a lethal model of fulminant liver failure induced by intraperitoneal injection of D-galactosamine and lipopolysaccharide in SCID mice^13^. Importantly, these adult stem cells require no genetic manipulations for their derivation, and are currently being employed in an AIFA (Agenzia Italiana del Farmaco)-approved Phase I clinical study in pediatric patients with inborn errors of metabolism at the Liver Transplant Center of the AOU Città della Salute e della Scienza in Turin, Italy (European Clinical Trials Database (EudraCT number: 2012-002120-33, https://eudract.ema.europa.eu/)). HLSC have been recognized by the European Medical Agency (EMA) as orphan drug for urea cycle disorders and acute liver failure and hence offer great promise for the treatment of other metabolic diseases such as CNSI.

In order to test the efficacy of HLSC in treating CNSI, we induced the differentiation and maturation of these cells in mouse liver scaffolds *ex vivo* and *in vivo* to assess whether these cells express UGT1A1. We generated a new immune-compromised mouse model (NOD SCID IL2Rgamma, NSG) bearing a mutation in the Ugt1 locus, capable of receiving human cells without the necessity of immune suppression to prevent graft rejection, to study the engraftment and function of the HLSC *in vivo*. We show herein that UGT1A1 enzyme expression and activity were recovered upon injection of a single dose of HLSC in NSG/Ugt1^−/−^ mouse livers *in vivo* resulting in an improvement of the phenotype characteristic of CNSI. Our study is a proof-of -concept study designed to test whether HLSC can express UGT1A1 *ex vivo* and *in vivo*. Results obtained in this short term study with a single HLSC injection are encouraging and warrant further studies on longer term.

## Results

### Characterisation of HLSC

**Figure 1:**
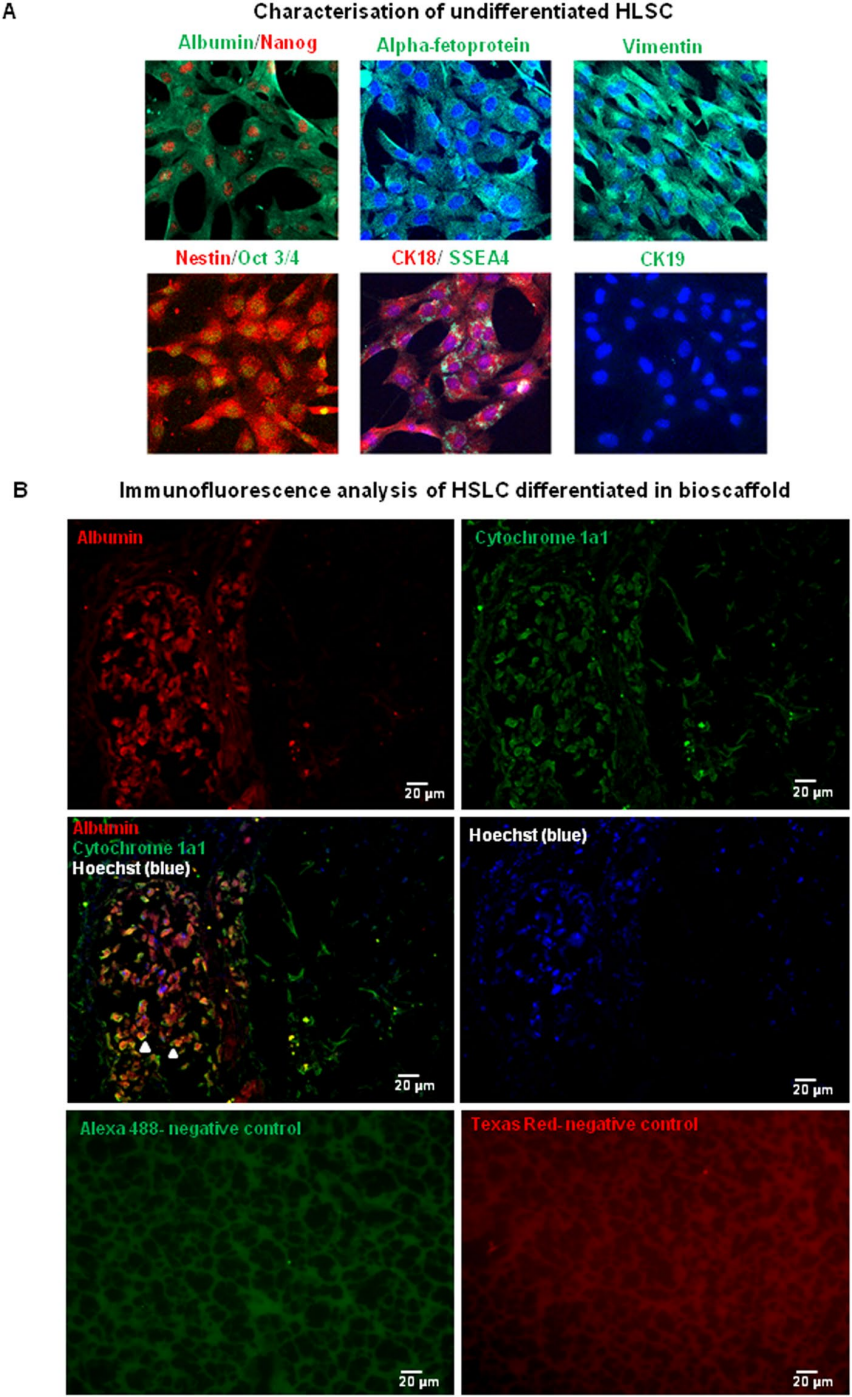
A Characterisation of HLSC. (**A**) Undifferentiated HLSC grown in chamber slides were stained for immunofluorescence analysis of Albumin, Alpha-fetoprotein, Vimentin, Nestin, Oct4, SSEA-4, CK18, CK19. Nuclei were stained with DAPI. Secondary antibody controls are shown. (**B**) Immunofluorescence analysis of HLSC differentiated in bioscaffold. HLSC differentiated for 15 days in bioscaffold were stained for the presence of the hepatocyte-specific markers, Albumin and Cytochrome 1a1. Nuclei were stained with Hoechst. Arrowheads show double-positive cells. Secondary antibody controls are shown.

HLSC were isolated and cultured as previously described^12^. The cell line HLSC-6b (wildtype for UGT1A1) was used in the present study, which shared characteristics with other two cell lines (HLSC-2 and HLSC-16, Table 1). These cells express hepatocyte-specific markers such as Albumin, alpha-fetoprotein and CK18, the mesenchymal marker, Vimentin, as well as the pluripotency markers Oct4 and SSEA4 (Fig. 1A). CK19 (a cholangiocyte marker) was not expressed in these cells (Fig. 1A).

**Table 1:** Characterisation of HLSC lines obtained from 3 independent donors.

HLSC-6b: from frozen hepatocyte (Lonza) (male, 15 year old)			
Markers	**Passage 2**	**Passage 6**	**Passage 12**
CD29	100%	100%	100%
CD73	98%	96%	87%
CD105	83%	91%	89%
Albumin	93%	91%	98%
**HLSC-2: from frozen hepatocyte (Lonza) (male, 70 year old)**			
Markers	**Passage 2**	**Passage 7**	**Passage 11**
CD29	98%	99%	100%
CD73	95%	91%	97%
CD105	93%	89%	94%
Albumin	95%	89%	97%
**HLSC-16: from frozen hepatocyte (Lonza) (male, 16 year old)**			
Markers	**Passage 2**		
CD29	100%		
CD73	99%		
CD105	98%		
Albumin	87%		

Percentage of HLSC expressing mesenchymal markers CD29, CD73 and CD105 and hepatocyte-specific marker Albumin was analysed by flow cytometry.

### HLSC differentiate into hepatocyte-like cells and express UGT1A1 protein *ex vivo*

**Figure 2:**
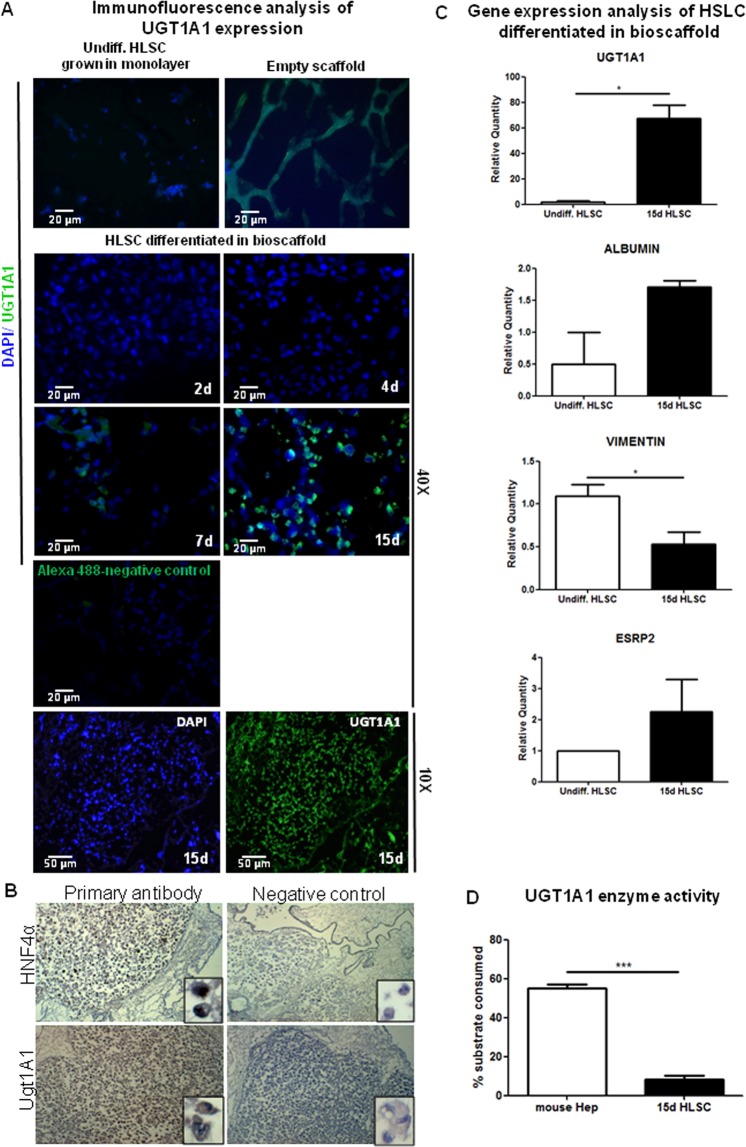
Analysis of UGT1A1 expression *ex vivo* in mice liver bioscaffolds. (**A**) Immunofluorescence analysis of HLSC differentiation in mice liver scaffolds at the indicated time points (days, d) showing human UGT1A1 expression (Magnification: 40X for all time points and 10X for 15d, n = 3). Undifferentiated (Undiff.) HLSC grown in monolayer, empty scaffolds and staining with secondary antibody only were used as controls. (**B**) Immunohistochemical analysis of differentiated HLSC showing expression of HNF4α (nuclear) and UGT1A1 (cytoplasmic). (**C**) Representative qRT-PCR analysis of mesenchymal marker (vimentin), hepatic marker (albumin, Ugt1a1) and epithelial marker (ESRP2) expression upon hepatocytic differentiation of HLSC at different time points. (**D**) UGT1A1 enzyme activity measured in HLSC cultured for 15 days in bioscaffolds *versus* wt mouse liver (n = 3).

We first assessed whether HLSC could express UGT1A1. HLSC induced to differentiate into hepatocyte-like cells in a rotary cell culture system expressed detectable mRNA levels of UGT1A1 as well as albumin, with respect to undifferentiated cells (Fig. S1). However, UGT1A1 protein was barely detectable using this system. We thus used an *ex vivo* differentiation system which further enhances HLSC differentiation and maturation. Mouse livers were decellularised and the bioscaffolds were repopulated with HLSC as we previously reported^14^. The scaffolds were maintained for 15 days to evaluate hepatocytic differentiation as shown by Albumin and CYP1A1 expression (Fig. 1B) as well as CYP7A1 and LDH (Fig. S2A). UGT1A1 expression was also evaluated at different time points after cell infusion into the bioscaffolds. Immunofluorescence analysis on mouse liver bioscaffolds revealed that HLSC expressed the enzyme as from 7 days of differentiation with a substantial increase after 15 days (Fig. 2A,B). Importantly, after 15 days of differentiation in the bioscaffolds, 63.4 ± 13.4% (mean ± standard error of mean; number of fields: 5) of HLSC expressed UGT1A1. Empty scaffolds and undifferentiated HLSC were used as negative control. Differentiated HLSC also expressed HNF4α confirming hepatocytic differentiation(Fig. 2B). In order to verify whether upon differentiation into hepatocyte-like cells, HLSC undergo Mesenchymal to Epithelial Transition (MET), the expression of MET-related genes were analysed. We found that there was a statistically significant decrease not only in Vimentin expression, but also in Snail and TGFB1 expression, which are known regulators of EMT, upon HLSC differentiation in liver scaffolds (Fig. S2B). The expression of ESRP2, which is an epithelial gene, showed an increase after 15 days of HLSC differentiation in decellularised mouse scaffolds (Fig. 2C), and is expected to increase further as HLSC mature into hepatocytes^15^. E-cadherin expression was undetectable in these cells (Fig. S2C). Thus, we could observe a partial MET trend in these cells upon hepatocyte differentiation. Importantly, bilirubin conjugation activity was also detected at 15 days of differentiation (Fig. 2D). The data revealed that HLSC differentiated *ex vivo* have 14.7% of UGT1A1 activity (8.1% of consumed substrate) with respect to wt mouse hepatocytes (100%; 55.1% of consumed substrate) concordant with previous studies showing that cell lines bear lower UGT enzyme activity *versus* mouse hepatocytes or primary hepatocytes^16^.

### HLSC engraft in NSG wt mouse livers *in vivo*

**Figure 3:**
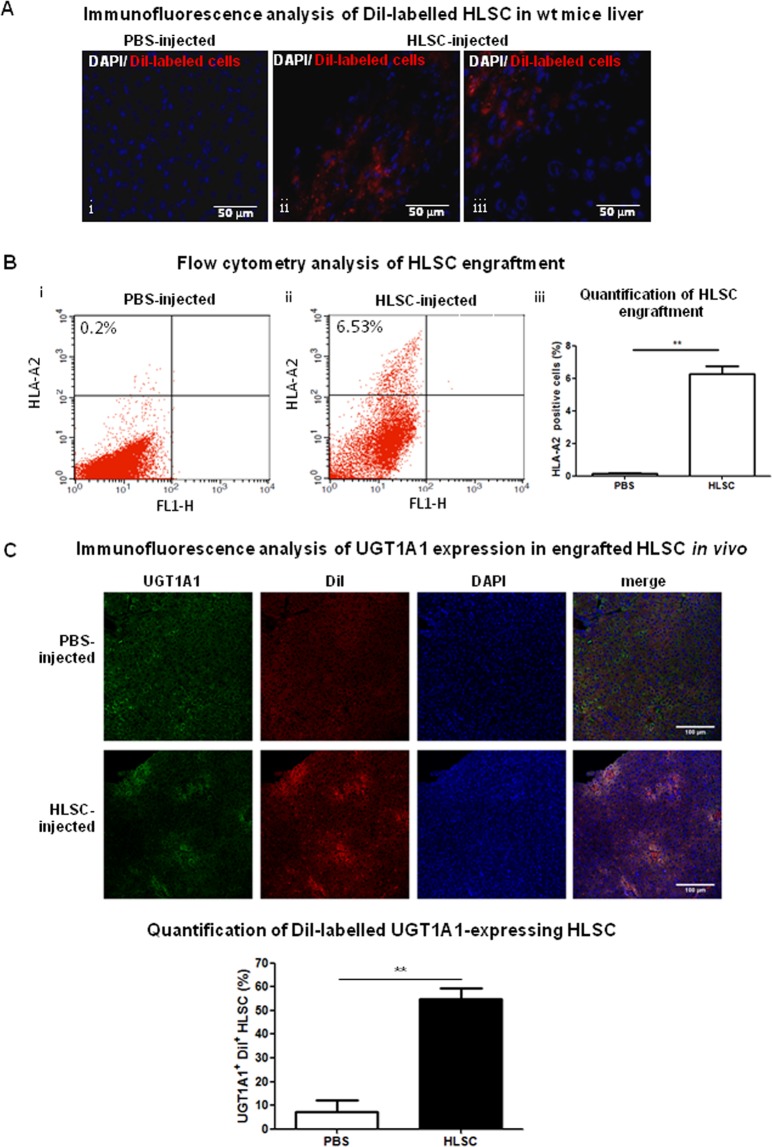
HLSC injection in NSG wt mice. (**A**) Immunofluorescence analysis of DiI-labelled non-injected control liver is shown in i; DiI-labelled HLSC in a 2 month old wt mouse liver after partial hepatectomy (ii, iii). (**B**) Flow cytometry analysis of 21 days old NSG mouse livers injected with (i) PBS and (ii) HLSC at Day 5. Each panel is representative of three independent experiments (n = 3). (iii) The graph shows percentage of HLSC engraftment (n = 3). (**C**) Liver of 21 days old pups injected at Day 5 either with PBS or with DiI-labelled (red) HLSC and stained for UGT1A1 (Alexa488, green) and DAPI (blue) (for higher magnification see Fig. S3) (*upper panel*). Quantification of the percentage of UGT1A1 expressing cells in the DiI positive population (*lower panel*); HLSC injected liver not stained for UGT1A1 was used as control. (n = 3).

To analyse HLSC engraftment *in vivo* in a NSG background, 2 months old wt mice were injected intraparenchymally after partial hepatectomy with DiI-labelled HLSC, and five days later, DiI-positivity was observed in the liver (Fig. 3A). To further evaluate the percentage of HLSC that colonize the liver of NSG pups, mice were injected with 1 × 10^5^ cells at 5 days after birth. Whole liver was then analysed in 21 days old mice (16 days after injection) using anti-HLA-A2 antibodies that reveal the presence of human cells in mouse liver. Interestingly, an average of 6.27 ± 0.83% of liver cells was positive for HLA-A2 compared to PBS-treated controls (Fig. 3B). These results were confirmed by injecting DiD-positive HLSC in the liver of mice and following cell engraftment over time (see Suplementary Methods and Fig. S3A). DiD-positive cells showed human HNF4α expression 15 days after cell injection (Figs. S3B and S4A,B). Moreover, immunofluorescence staining of liver sections of HLSC-injected mice for UGT1A1 revealed that 54.74 ± 4.65% (mean ± standard error of mean; number of mice: 3; number of fields: 7) of DiI-positive cells expressed the enzyme *in vivo* compared to 7.16 ± 4.00% in control livers (Figs. 3C and S5). DiI-positive cells were located around sinusoids, showing that HLSC engrafted in mouse livers (Fig. S5).

### HLSC injection prolongs NSG/Ugt1^−/−^ mice survival by expressing UGT1A1

**Figure 4:**
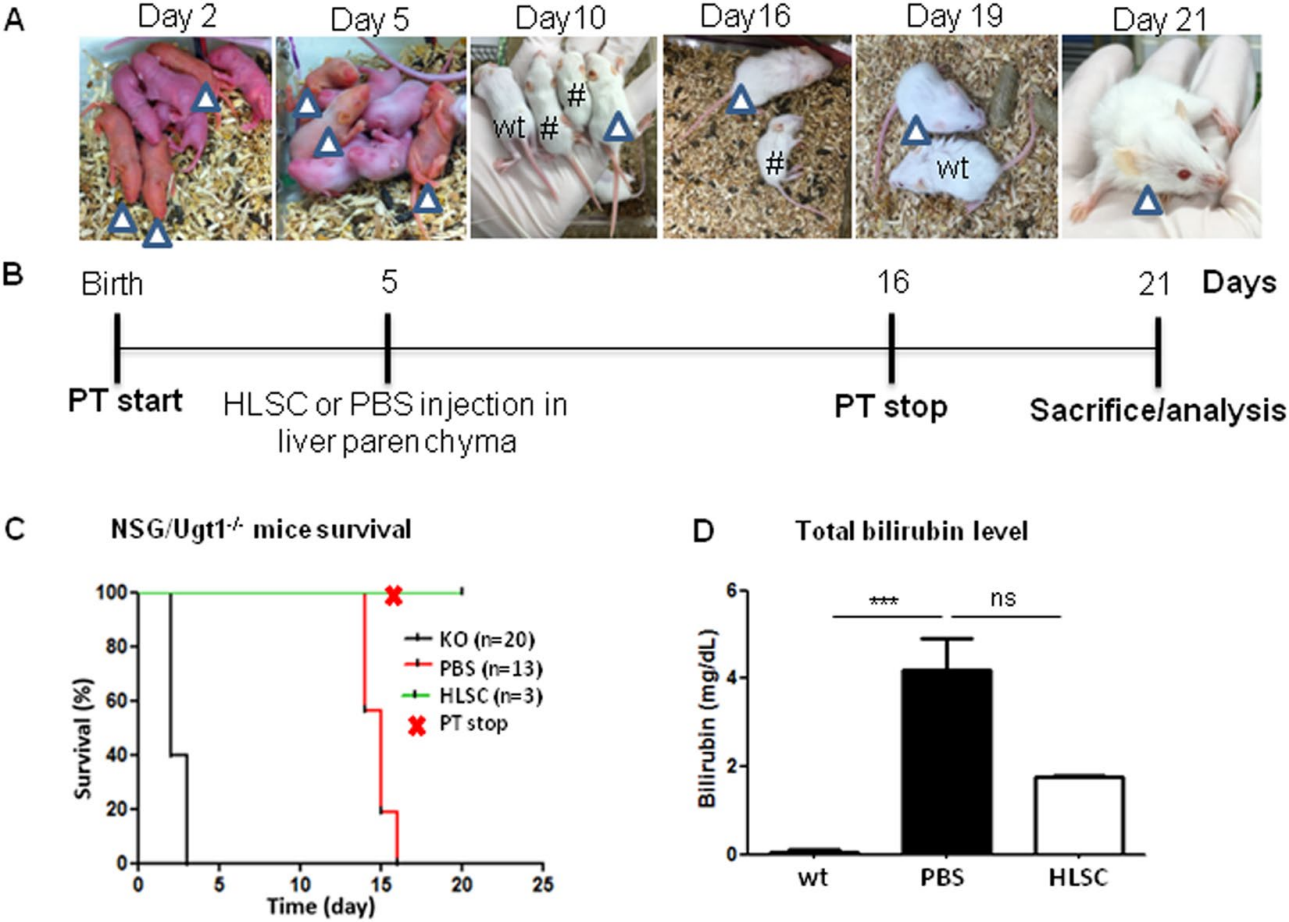
Mice survival and HLSC functionality assessment after cell injection in NSG/Ugt1^−/−^mice. (**A**) Photographs of NSG/Ugt1^−/−^ mice treated with HLSC or PBS. NSG/Ugt1^−/−^ mice (arrowheads) at neonatal Day 2 and Day 5. PBS-injected mice (#) and HLSC-injected mouse (arrowheads) compared to a wt mouse (wt) at Day 10. HLSC-injected mouse (arrowhead) at Day 19 and Day 21 post-injection. (**B**) Schedule of HLSC or PBS injection in PT-treated NSG/Ugt1^−/−^ mice. (**C**) Survival of NSG/Ugt1^−/−^ mice until Day 21 is shown (NSG/Ugt1^−/−^ without PT (KO, n = 20) *versus* NSG/Ugt1^−/−^ PT-treated mice, injected with PBS (n = 13) or HLSC (n = 3)). (**D**) Total bilirubin level in mouse serum: wt (n = 2) *versus* Day 8 PBS-treated (n = 5) and Day 21 HLSC-treated (n = 2) NSG/Ugt1^−/−^ mice (ns is non-significant using Bonferroni Multiple comparison test).

We further explored the efficacy of HLSC in restoring functionality in a CNSI model *in vivo*. To this purpose, we generated an immune-compromised mouse model of the Crigler Najjar syndrome by backcrossing Ugt1^+/−^ with NSG mice for 7 generations to derive NSG/Ugt1^−/−^ mice (see Suplementary Methods and Fig. S6). NSG/Ugt1^−/−^ mice had the visible appearance of jaundice (by postnatal Day 2), as evidenced by orange skin colour (Figs. 4A and S6). The HLSC treatment schedule is shown in Fig. 4B. NSG/Ugt1^−/−^ pups without PT survived for only 2 to 3 days after birth (Fig. 4C, KO: n = 20). PT was thus started at birth in specific-pathogen-free cages (Figs. 4B and S6).

In order to assess therapeutic utility of HLSC, cells or PBS were directly delivered in the liver parenchyma of postnatal Day 5 pups undergoing PT from birth. After HLSC or PBS injection, mice were maintained under PT. PBS-treated NSG/Ugt1^−/−^ mice did not survive beyond post-natal Day 16 (Fig. 4C, PBS: n = 13), had severe motility impairment and a significant reduction in body mass (Fig. 4A Day 16, and Movie S1). Thus, at Day 16, PT was removed from HLSC-injected mice and the efficacy of cell therapy in improving phenotype and survival was assessed. Interestingly, there was a 100% survival in NSG/Ugt1^−/−^ HLSC-treated groups up to Day 21(last time point analysed), despite PT removal, compared to PBS-treated mice (50% survival: 15 days) (Fig. 4C, HLSC: n = 3). Most importantly, HLSC-injected NSG/Ugt1^−/−^ mice analysed at 21 days were comparable to the wt littermates, with no apparent motor deficits, with respect to PBS-injected NSG/Ugt1^−/−^ mice (Fig. 4A, Day 19 and Movies S1 and S2). To analyse engraftment of HLSC, HLSC-treated mice were sacrificed at Day 21. Importantly, in these mice, total bilirubin levels decreased by two-fold compared to PBS-treated 8 days old ones used as controls (Fig. 4D). When compared to mutant mice in the FVB background, the PT-treated Day 8 NSG/Ugt1^−/−^ mice showed similar levels of total bilirubin as the PT-treated Day 8 FVB/Ugt1^−/−^ mice. Moreover, PT-treated FVB/Ugt1^−/−^ mice total bilirubin level increased significantly from Day 8 to Day 18 (Fig. S7). Thus, the bilirubin level of PT-treated Day 18 FVB/Ugt1^−/−^ mice was compared to that of the Day 21 HSLC-injected NSG/Ugt1^−/−^ mice. Interestingly, the HLSC-treated mice showed a significantly lower total bilirubin level compared to the Day 18 FVB/Ugt1^−/−^ control mice (Fig. S7).

**Figure 5:**
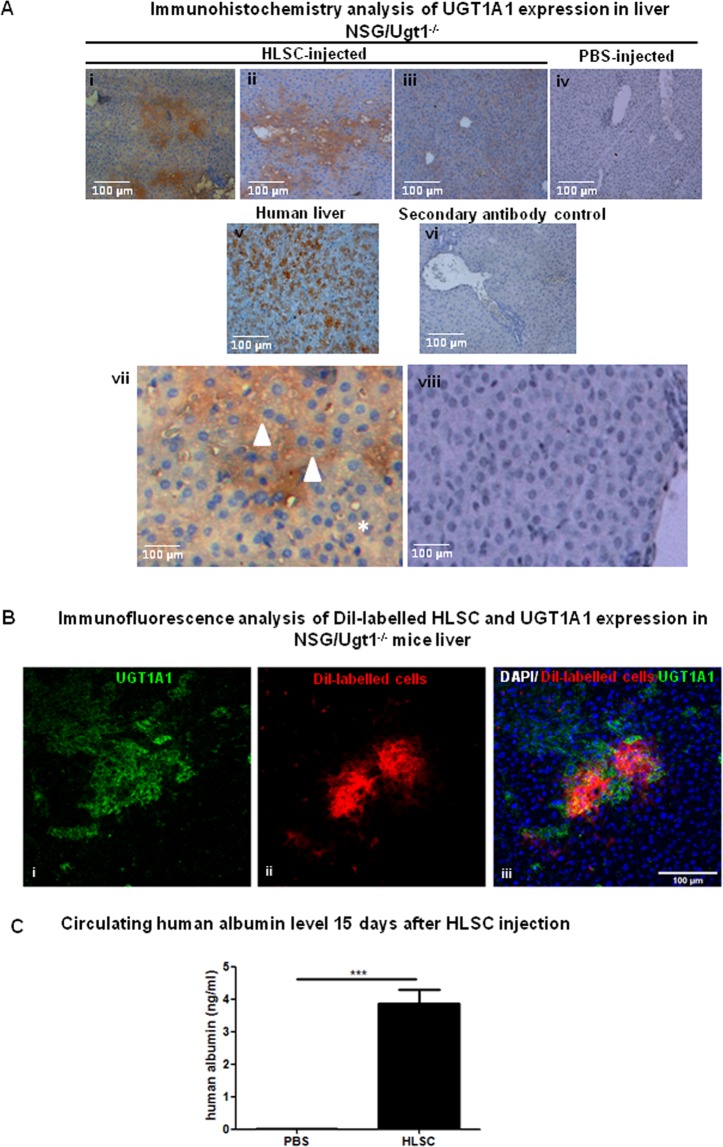
HLSC tracking in NSG/Ugt1^−/−^ mice. (**A**) Immunohistochemistry analysis of UGT1A1 expression *in vivo* in 21 days old mouse livers. UGT1A1-positive (i, ii) and UGT1A1-negative (iii) areas in HLSC-injected NSG/Ugt1^−/−^ mouse livers are shown. PBS-injected NSG/Ugt1^−/−^ (iv) mice and human liver sections (v) were used as negative and positive controls, respectively. Zoomed image of the HLSC-injected (vii, from i) and PBS-injected (viii, from iv) mouse livers show positivity of Ugt1a1 in binuclear hepatocyte cytoplasm of HLSC-injected mice liver, with respect to the absence of signal in the PBS-injected control. (**B**) Immunofluorescence analysis of 21 days old HLSC-treated NSG/Ugt1^−/−^ mouse livers showing DiI (i)- and UGT1A1 (ii)- positivity; merged images in (iii). Inset shows DiI-positive HLSC (red) and UGT1A1 (green) colocalization (yellowish, arrowheads). (**C**) Human Albumin level in serum was measured by ELISA in PBS- and HLSC-injected mice.

In order to assess the degree of UGT1A1 expression and cell grafting in HLSC-injected NSG/Ugt1^−/−^ mice, liver sections from treated mice were analysed by immunohistochemistry with anti-UGT1A antibodies. As shown in Fig. 5A, HLSC showed regional and heterogeneous engraftment in the injected lobe as expected after intraparenchymal injection of cells^17^. There was an intense HLSC-induced UGT1A1 expression in the liver of HLSC-injected NSG/Ugt1^−/−^ mice compared to PBS-injected controls. (Fig. 5A i, ii, and iii *versus* iv, respectively). As expected when cells are injected directly in the parenchyma, engraftment was regional and heterogeneous in the injected liver lobes^17,18^. Human liver sections, used as positive control, showed diffuse UGT1A1 staining compared to the localized staining in HLSC-injected NSG/Ugt1^−/−^ mice (Fig. 5A v *versus* i, ii, iii, respectively). UGT1A1 staining was specific to binuclear hepatocyte-like cells (Fig. 5A vii) compared to the controls (Fig. 5A viii). DiI-positivity and UGT1A1 immunoreactivity colocalised in the liver of DiI-stained HLSC-injected NSG/Ugt1^−/−^ mice (Fig. 5B). The DiI-positive cells were negative for the proliferation marker PCNA suggesting that HLSC do not proliferate at the time point analysed (15 days after cell injection) (Fig. S8). Importantly, human albumin was detected in mouse serum 15 days after cell injection (Fig. 5C). These data show that injected cells engrafted in NSG/Ugt1^−/−^ mouse livers *in vivo* and induced UGT1A1 expression. No cell fusion events were observed between human and mouse cells in this model 15 days after HLSC injection (See Supplementary methods and Fig. S9).

### HLSC injection reduces brain injury in NSG/Ugt1^−/−^ mice

**Figure 6:**
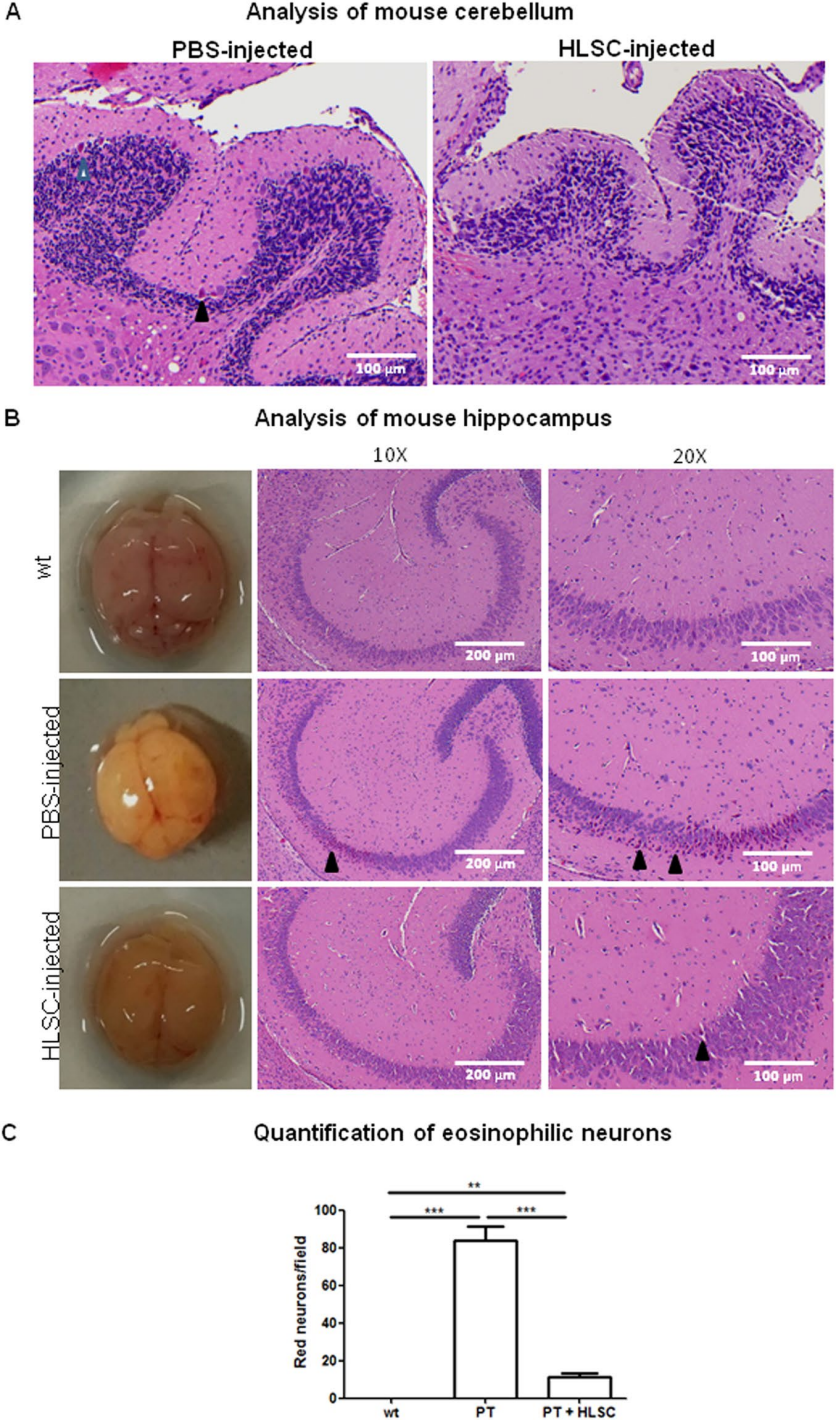
Analysis of mice brain. (**A**) H/E-stained sections of HLSC-injected NSG/Ugt1^−/−^ mice (n = 3) cerebellum compared to PBS-injected controls (n = 3). Arrowheads show eosinophilic neurons. (**B**) Representative image of brains and H/E-stained hippocampal sections of HLSC-injected NSG/Ugt1^−/−^ mice compared to wt and PBS-injected NSG/Ugt1^−/−^ controls. Arrowheads show eosinophilic neurons. (**C**) Quantification of eosinophilic neuron on 3 fields/section (3 sections/mouse, n = 3).

Brain damage in the cerebellum (Fig. 6A) and hippocampus (Fig. 6B) of the NSG/Ugt1^−/−^ mice, evidenced by the number of eosinophilic neurons (Fig. 6B, arrowheads and Fig. 6C), was reduced upon HLSC injection with respect to PBS-treated controls, showing that these cells efficiently prevented the pathological effects of unconjugated bilirubin when injected early in the newborns^19^. For serum bilirubin and brain and liver histology assessment, 21 days old HLSC-injected NSG/Ugt1^−/−^ mice could not be compared with 21 days old control NSG/Ugt1^−/−^ mice as the latter did not survive beyond 16 days despite PT.

## Discussion

The use of autologous stem cells to correct liver function in monogenic liver diseases is considered to be a very promising approach^20^. As no HLA matching is required for cell transplant in the liver, ample donor-derived stem cells can be employed for cell therapy^21^. We thus explored whether cell therapy with HLSC, which are stem cells isolated from healthy human liver tissue, is capable of rescuing CNSI phenotype in immune-compromised Ugt1^−/−^ mice.

For this study, we generated Ugt1^−/−^ mice in NSG background which better support human stem cell engraftment compared to other immune-compromised mice such as SCID^22^. Cells were injected in our model at postnatal Day 5 when the liver is growing. The highly proliferative state of the liver in very young animals enhances the effectiveness of cell therapy in the neonatal/pediatric period and may prevent tissue damage that may not be possible to correct later in life^23^. Importantly, around 5% of replacement of the total liver mass may improve metabolic disorders, and 10% may normalize liver function^24^. For instance, in humans, infusion of 5% of the liver mass efficiently lowered bilirubin levels in CNSI patients^10,25,26^. In our model, by directly delivering a single dose of HLSC to newborn NSG wt mouse livers, we found an average of 6.27 ± 0.83% of engrafted human cells 15 days post-injection. The potency of HLSC was proven in *ex vivo* culture as well as by *in vivo* differentiation after transplantation in mice by their ability to express UGT1A1 enzyme and to lower circulating bilirubin levels. Importantly, bilirubin conjugation activity was observed in HLSC differentiated in bioscaffolds *versus* wt mouse hepatocytes (14.7% versus 100%, respectively), concordant with previous studies showing that cell lines bear lower UGT enzyme activity compared to the whole liver or primary hepatocytes^16^. Maturation of HLSC into hepatocyte-like cells *in vivo* is observed as early as 15 days after cell injection as shown by our data on UGT1A1 expression in the liver of knockout mice. Injected HLSC did not proliferate 15 days after injection in mice liver, suggesting that cells had differentiated. The amount of human Albumin (around 4 ng/ml) detected in mouse serum 15 days after cell injection was consistent with the single injection of 1 × 10^5^ cells in one liver lobe compared to other studies using a higher number of cells^9^. As neurons are sensitive to hyperbilirubinemia, we analysed the mouse cerebellum and hippocampus for the presence of dying neurons. Strikingly, brain damage and presence of eosinophilic neurons, mainly in the hippocampus of the NSG/Ugt1^−/−^ mice, were reduced upon HLSC injection with respect to PT-treated controls. Red neurons are representative of ischemic and hypoxic injury, which may be induced by unconjugated bilirubin toxicity in PBS-injected NSG/Ugt1^−/−^ mice, leading to death within 16 days after birth^27^. On the other hand, HLSC-injected NSG/Ugt1^−/−^ mice at 21 days after birth had no apparent motor deficits compared to their PBS-injected counterparts, showing that these cells efficiently prevented the pathological effects of unconjugated bilirubin when injected early in the newborns (Movie S2)^19,28,29^.

Therapies based on gene and cell transfer techniques are mostly in the preclinical phase. It is crucial to evaluate the safety, potency, absence of tumorigenicity and toxicity, as well as *in vivo* biodistribution of the cells in animal models before their clinical development^24^. Cell-free, AAV-mediated gene therapy for CNSI has proved to be very effective in rescuing bilirubin-induced neonatal lethality in Ugt1^−/−^ mice and Gunn rat^30–32^. Cell therapy has been mainly conducted in the Gunn rat^9^. The immune-compromised NSG/Ugt1^−/−^ mice may also offer a platform for *in vivo* testing of safety and efficacy of human cells^33^. These mice offer the advantage of being lethal, hence better mimicking the human disease, if therapy is not efficiently delivered early after birth.

Importantly, a very recent study evaluated the safety of using human liver-derived stem cells, termed Heterologous Human Adult Liver-derived Progenitor Cells (HHALPCs), in CNSI patients in a phase I/II prospective trial^34^. During the treatment period, patients were subjected to phototherapy daily. HHALPCs delivery induced a ~ 20% decrease in bilirubin levels in 2 patients, one of whom (a female) showed successful liver engraftment of male stem cells. A transient effect was observed in another CNSI patient^34^. This study demonstrates the overall safety of liver-derived adult stem cells injection in CNSI patients, as well as in other severe metabolic liver diseases such as urea cycle disorder. Our data, using HLSC injection in mice, further support, at a preclinical level, the clinical study performed by Smets *et al*., and show the great opportunity presented by human liver stem cells, derived using different approaches, in resolving the problem of scarcity of liver donors for the treatment of severe liver diseases.

Scalable production of HLSC in GMP (Good Manufacturing Process) is now possible and has allowed performing the first-in-man Phase 1 clinical study in neonates with urea-cycle disorders (EudraCT number: 2012-002120-33). Our results show that a single HLSC injection ameliorates the phenotype and survival of NSG/Ugt1^−/−^ mice by differentiating into UGT1A1-expressing hepatocyte-like cells with UGT1A1 enzyme activity. Future long-term studies are needed to assess whether multi-dose injection of HLSC will further lower the level of unconjugated bilirubin in NSG/Ugt1^−/−^ mice.

## Methods

### Cell culture and differentiation

The isolation and characterisation of HLSC (proprietory cell line to Unicyte) have been previously described^12^. HLSC-6b cell line was cultured in α-MEM/EBM-1 (3:1) (Invitrogen, Carlsbad, CA, USA) media supplemented with L-glutamine (5 mM), Hepes (12 mM, pH 7.4), penicillin (50 IU/ml), streptomycin (50 μg/ml) (all from Sigma-Aldrich) and fetal calf serum (FCS) (10%) (Invitrogen)^35^. Cells were analysed for Albumin expression by immunofluorescence. HLSC (2.5 × 10^6^) were differentiated in a rotary cell culture system (RCCS) in standard medium as previously described in order to analyse hepatic gene expression^12^. To further promote the differentiation of HLSC into hepatocyte-like cells, the cells were seeded into mice acellular liver bioscaffolds as previously described, and UGT1A1 expression was assessed at different time points^14^. Briefly, 80 × 10^6^ HLSC, cultured for at most 6 passages, were infused in the bioscaffolds using the standard HLSC culture medium and maintained at 37 °C and 5% CO_2_ for 15 days. The medium was changed every other day during the entire experiment. Differentiation of HLSC into hepatocyte-like cells was verified by immunofluorescence staining for Albumin (MAB1455, R&D Systems), Cytochrome 1A1 (CYP1A1; ab79819, Abcam), Cytochrome 7A1 (CYP7A1; ab65596 Abcam), Lactate dehydrogenase (LDH; ab52488, Abcam) and Vimentin (ab16700, Abcam), and revealed by Alexa488- or Texas Red-labelled secondary antibodies (Abcam). Human UGT1A1 expression was analysed by immunofluorescence using rabbit antibody against the protein (UGT1A, sc-25847, Santa Cruz Biotechnology) and was revealed using Alexa488-conjugated anti-rabbit secondary antibody (Invitrogen). Immunohistochemical analysis was performed using anti-human UGT1A1 (MBS9203265, MyBioSource) and HNF4α (ab92378, Abcam) antibodies as previously described^36^. Briefly, After antigen retrieval (for HNF4 α), sections were stained with primary antibodies and revealed with biotinylated anti-rabbit antibody and the ABC complex (Vector Labs) followed by exposure to 3, 3′-Diaminobenzidine (DAB, Roche).Nuclei were counterstained with hematoxylin.

### Generation of NSG/Ugt1^−/−^ mouse model and phototherapy

NSG/Ugt1^+/−^ mice were obtained by backcrossing our previously described C57Bl/6 Ugt1^+/−^ mice with NOD^*scid*^ IL2Rg^null^ (NSG) wild type (wt) (JAX 005557) mice for 7 generations^30,37^. For each generation, Ugt1^+/−^ mice were selected by genotyping for the NSG background (https://www.jax.org/strain/005557) and Ugt1a1 mutation as previously described^37^. Primers are listed in Table S1. NSG/Ugt1^−/−^ mice were then derived by crossing NSG/Ugt1^+/−^ mice. Mice were bred under specific-pathogen-free conditions and allowed free access to regular chow (standard diet 4RF21, Mucedola srl) and water. All animals received humane care according to the criteria outlined in “Guide for the Care and Use of Laboratory Animals, 8th edition” and institutional guidelines^38^. To perform PT under sterile conditions, we devised special cages with internal blue LED lights (450 nm) in order to deliver uniform irradiation (HLMP-CB3A-UV0DD, 20 Ma, 3.2 V, 450 nm, Avago Technologies). A timer connected to the cage ensured 14 hours’ daily exposure to blue light during the light cycle. This study was approved by the local ethical committee and the Italian Ministry of Health (number 1110/2015-PR).

### HLSC transplantation in mice

To verify the ability of HLSC to express UGT1A1 *in vivo*, DiI (1,1′-Dioctadecyl-3,3,3′,3′-Tetramethylindocarbocyanine Perchlorate, Thermo Fisher Scientific)-labelled cells were initially injected in 2 months old wt (wildtype) mouse livers after partial hepatectomy (removal of left lobe). Undifferentiated (1 × 10^5^ cells/30 µl PBS) HLSC were injected intraparenchymally in the median lobe which was taken after 15 days for immunofluorescence analysis.

All NSG/Ugt1^−/−^ pups underwent PT as from birth. A single dose of HLSC (1 × 10^5^) stained with DiI was injected directly in the liver parenchyma (still visible through the skin at this age) of 5 days old pups. PBS-injected mice were used as negative control. At Day 16 after birth (a time point beyond which no controls survived), PT was removed. HLSC-injected mice were left without PT for another 5 days, and phenotype rescue evaluations were performed at Day 21 after birth (16 days after cell injection).

### Serum bilirubin measurement

Serum bilirubin levels were measured in NSG/Ugt1^−/−^ mice injected with HLSC at Day 21 using Bilirubin, Total, kit (BQ kits, Inc.) as previously described^28^. PBS-treated NSG/Ugt1^−/−^ mice at postnatal day 8 and sibling littermate wt mice were used as positive and negative controls, respectively. We also compared the level of total bilirubin in the serum of NSG/Ugt1^−/−^ mice with that of FVB/Ugt1^−/−^ mice at postnatal Day 8 with and without PT and at Day 18 and Day 30 with PT.

### RNA extraction and gene expression analysis

RNA was extracted from HLSC differentiated in RCCS or bioscaffolds using the PureLink RNA kit (Ambion) and cDNA was prepared using the High-Capacity cDNA Reverse Transcription Kit (Applied Biosystems). Target gene expression was analysed by quantitative Real-time PCR (qRT-PCR) on ABI 7300 Real-time system and normalized to endogenous 18S rRNA expression (4319413E, ThermoFisher Scientific) as previously described^17^. Universal ProbeLibrary probes were used for target gene expression analysis (Roche). Primers used for qRT-PCR are listed in Table S1.

### Western blotting

Total protein was extracted from HLSC differentiated in RCCS, from human hepatocytes (Lonza) or mouse primary hepatocytes using lysis buffer containing 1% Triton X-100 supplemented with protease inhibitors (Complete Mini, Roche) and 50 microgram samples were separated by 4–15% SDS-PAGE (Biorad). After protein transfer, nitrocellulose membrane was saturated with 5%BSA and incubated overnight at 4 °C with rabbit anti-Ugt1A antibody (Ugt1A, sc-25847, Santa Cruz Biotechnology) and mouse anti-vimentin (in-house). After washing, the membrane was incubated for one hour at room temperature with anti-rabbit and anti-mouse secondary antibodies from Sigma and developed using ECL (Biorad) on Chemidoc imaging system (Biorad). Densitometric analysis was performed using the volume analysis tool of ImageLab software (Biorad Laboratories Inc).

### UGT1A1 enzyme activity

Differentiated HLSC were assessed for UGT1A1 enzyme activity^39^. Briefly, mice liver bio-scaffolds were replenished with HLSC (80 × 10^6^ cells) and maintained in culture for 15 days as previously described^14^. The bioscaffolds were then pulverized in liquid nitrogen, and homogenized in ice-cold PBS. Following centrifugation to eliminate debris and nuclei, the resulting supernatant was centrifuged at 100 000 g for 60 min at 4 °C, as previously described^39^. The microsomal pellet was resuspended in buffer (50 mM Tris-HCl, pH 7.4, 10 mM MgCl_2_, 1 mM phenylmethylsulfonyl fluoride), and the protein concentration was determined by the Bradford method. UGT1A1 activity was then measured using 0.2 mg/ml protein and UGT Glo assay following the manufacturer’s protocol (Promega) and as previously described^40^. Microsomes derived from wt mouse hepatocytes were used as positive control.

### Immunofluorescence, immunohistochemistry and histology of mouse tissues

Liver, brain and blood were taken at indicated time points. Liver lobes of Day 8 wt and PBS-treated NSG/Ugt1^−/−^ mice as well as Day 21 HLSC-treated NSG/Ugt1^−/−^ mice were fixed in formalin, included in paraffin and processed for immunofluorescence studies. UGT1A1 expression was analysed by using rabbit antibody against the protein (UGT1A, sc-25847, Santa Cruz Biotechnology; anti-human UGT1A1, MBS9203265, MyBioSource) and was revealed using Alexa488-conjugated anti-rabbit secondary antibody (ThermoFisher Scientific). Anti-PCNA (sc-56, Santa Cruz Biotechnology) was also used to detect proliferating cells and was revealed using Alexa488-conjugated anti-mouse secondary antibody. DiI-labelled cells were tracked using a Zeiss microscope and Apotome software. UGT1A1 and DiI colocalisation was performed using Leica TCS SP5 confocal system (Leica Microsystems) equipped with a 405 nm diode, an argon ion, a 561 nm DPSS and a HeNe 633 nm lasers. Liver sections were imaged using a 40×/1.25 NA oil immersion objective. Formalin-fixed, paraffin-embedded liver sections were stained with haematoxylin/eosin. For immunohistochemistry, liver sections were stained with anti-UGT1A antibody and revealed with biotinylated anti-rabbit antibody, the ABC complex (DAKO) and 3,3′*-*Diaminobenzidine (DAB, Roche). Brain sections were also stained with haematoxylin/eosin.

### Flow cytometry

HLSC engraftment was assessed by injecting the cells in the liver of 5 days old NSG wt mice and analyzing the percentage of HLSC present after 16 days of cell injection using PE Mouse anti-Human HLA-A2 (clone BB7.2, BD Pharmigen™) by flow cytometry. Briefly, mouse livers were perfused with Liver Perfusion solution followed by Liver Digest medium as per manufacturer’s protocol (ThermoFisher Scientific). Cells were stained with anti-HLA antibodies and data were analysed using the CellQuest software (BD FACSCalibur).

### Human albumin ELISA

A human-specific Albumin ELISA kit (ab179887) was employed for assessing the circulating levels of human Albumin in HLSC-injected mice, and was performed according to the manufacturer’s instructions (Abcam).

### Statistical analyses

Data are expressed as mean ± standard deviation (s.d) and are representative of at least 3 independent experiments. Statistical differences (where n > 3) were determined by a 2-tailed Student’s *t* -test (*P < 0.05, **P < 0.01, ***P < 0.001) or One-way ANOVA (*P < 0.05, ***P < 0.001) and Bonferroni Multiple comparison test. All experiments were performed independently at least 3 times, unless indicated otherwise. Number of mice employed in each experiment is indicated the figure legends.

### Ethical approval and informed consent

This study was approved by the local ethical committee and the Italian Ministry of Health (number 1110/2015-PR). All experiments were performed in accordance to the criteria outlined in “Guide for the Care and Use of Laboratory Animals, 8th edition” and institutional guidelines.

## Supplementary information

Supplementary Information.

Movie S1.

Movie S2.

## Data Availability

No datasets were generated or analysed during the current study.
