# Supplementary material for: Human liver stem cells express UGT1A1 and improve phenotype of immunocompromised Crigler Najjar syndrome type I mice
Source: Sci Rep. 2020 Jan 21;10:887. doi: 10.1038/s41598-020-57820-2 (PMC6972964; doi:10.1038/s41598-020-57820-2)
Supplement: Supplementary file 1 — Supplementary Information. [file 41598_2020_57820_MOESM1_ESM.zip › Supplementary Information/Famulari et al_Table S1.docx]

| **Primers for PCR** | | |
| --- | --- | --- |
| **Name** | **5' to 3' sequence** | **References** |
| Ugt rev | GCTGTAAGACAATCTTCTCC | ^30^ |
| Ugt fw | TCACCAGAGTAGGCATCTC |  |
| **Primers for qRT-PCR** | | |
| **Name** | **5' to 3' sequence** | **Roche probe^/reference^** |
| hUgt1A | atatggtttttgttggtggaatc  gcattaatgtaggcttcaaattcct | #8 |
| hVimentin | TACAGGAAGCTGCTGGAAG ACCAGAGGGAGTGAATCCAG | #13^/36^ |
| hAlbumin | aatgttgccaagctgctga cttcccttcatcccgaagtt | #27 |
| hHNF4a fw | gttgacgatgggcaatgac cgaggcaccgtagtgtttg | #64 |
| hTGFb fw | ACTACTACGCCAAGGAGGTCAC TGCTTGAACTTGTCATAGATTTCG | #31 |
| hSnail fw | GCTGCAGGACTCTAATCCAGA ATCTCCGGAGGTGGGATG | #11^/36^ |
| hESRP2 fw | gctgttatcctccatctactcaaag gtccaccacatcagccttg | #3 |
| hE-cadherin | CCCGGGACAACGTTTATTAC GCTGGCTCAAGTCAAAGTCC | #35^/36^ |
