# Supplementary material for: Human liver stem cells express UGT1A1 and improve phenotype of immunocompromised Crigler Najjar syndrome type I mice
Source: Sci Rep. 2020 Jan 21;10:887. doi: 10.1038/s41598-020-57820-2 (PMC6972964; doi:10.1038/s41598-020-57820-2)
Supplement: Supplementary file 1 — Supplementary Information. [file 41598_2020_57820_MOESM1_ESM.zip › Supplementary Information/Famulari et al_Table S2.docx]

| **Pups Genotype** | **Treatment** | **Number** | **Maximum Survival Days** |
| --- | --- | --- | --- |
| Wildtype | None | 15 | 21 (end-point) |
| NSG/Ugt1^-/-^ | None | 20 | 3 |
| NSG/Ugt1^-/-^ | Phototherapy and PBS | 13 | 16 |
| NSG/Ugt1^-/-^ | Phototherapy and HLSC | 3 | 21 |

Table S2: Mice used in the survival study
