# Supplementary material for: Human liver stem cells express UGT1A1 and improve phenotype of immunocompromised Crigler Najjar syndrome type I mice
Source: Sci Rep. 2020 Jan 21;10:887. doi: 10.1038/s41598-020-57820-2 (PMC6972964; doi:10.1038/s41598-020-57820-2)
Supplement: Supplementary file 1 — Supplementary Information. [file 41598_2020_57820_MOESM1_ESM.zip › Supplementary Information/Supplementary Legends.docx]

**Figure S1: I. Differentiation of HLSC in rotary cell culture system (RCCS) *in vitro***. A. QRT-PCR analysis of UGT1A1 expression in undifferentiated HLSC *versus* human hepatocytes. B. Western blot analysis of UGT1A1 protein in mouse liver (m-liver) and human hepatocytes (h-Hep) *versus* undifferentiated HLSC. C. The rotary cell culture system (RCCS) showing aggregates of differentiating HLSC (arrowheads). D. QRT-PCR analysis of vimentin, albumin and UGT1A1 in HLSC during hepatocytic differentiation.

**Figure S2: HLSC differentiated in mouse decellularised liver scaffold.** A. Sections of scaffolds were co-stained for Albumin and Cytochrome 7a1 (Cyp7a1) or lactate dehydrogenase (LDH) or Vimentin. B. Real-time PCR analysis of EMT-related gene expression. C. Immunohistochemical analysis of E-cadherin expression in undifferentiated (Undiff.) and differentiated (Scaffold 15d) HLSC compared to HaCAT cells, used as positive control.

**Figure S3: HLSC injection in mice livers and control of cell engraftment.** A. Representative images obtained by optical imaging of 5 days old pups or adults after intrahepatic injection of DiD-positive cells. B. Flow cytometry analysis of HNF4α expression in DiD-positive HLSC isolated from mouse livers 15 days post-transplantation, compared to PBS-injected controls.

**Figure S4: Human HNF4α expression in mouse liver after HLSC injection.** A. Immunofluorescence analysis of liver sections shows co-localisation of HNF4α- and DiI-positivity. B. Real-time PCR analysis of HNF4α expression in mouse livers 15 days after HLSC transplantation compared to PBS-injected controls.

**Figure S5: HLSC injection in NSG wt mice.** Liver of 5 days old pups was injected either with PBS or with DiI-labelled (red) HLSC and stained for UGT1A1 (green) and DAPI (blue) 14 days after injection as in Figure 2C. In HLSC injected liver, DiI positive cells expressed UGT1A. B. Quantification of DiI-positive cells in mouse liver. Method for cell selection and quantification in 3 independent fields is shown. Graph shows percentage of Ugt1a1+/DiI+ cells from individual mouse liver, as well as the mean.

**Figure S6:** **Generation and characterisation of NSG/Ugt1^-/-^ mice**. A. Genotyping of mice by PCR (KO = knockout allele; wt = wild type allele). B. Analysis of UGT1A1 expression by western blot. C. Two days old litters showing NSG/Ugt1^-/-^ mice (yellowish, arrowheads). D. Five days old litters with NSG/Ugt1^-/-^ mice under phototherapy (PT) (yellowish, arrowheads). E. Sterile cages were devised with blue lights for PT of NSG/Ugt1^-/-^ mice.

**Figure S7: Comparison of total bilirubin (TB) levels between FVB/Ugt1^-/-^ and NSG/Ugt1^-/-^ mice**. A.Total Bilirubin levels in wt mice *versus* FVB/Ugt1^-/-^ mice at different postnatal days (P). B. Total Bilirubin levels in PT-treated FVB/Ugt1^-/-^ mice and NSG/Ugt1^-/-^ mice at postnatal Day 8. C. Total Bilirubin levels in FVB/Ugt1^-/-^ mice *versus* HLSC-treated NSG/Ugt1^-/-^ mice.

**Figure S8: PCNA staining and DiI-positivity in HLSC-injected mouse liver.** PCNA staining (green, Alexa488) is shown in PBS-injected and HLSC-injected mouse livers. DiI (red)-positive areas in HLSC-injected mice are also shown.

**Figure S9: FISH analysis of HLSC-injected mice-derived liver cells.** Human cells (Cy3-labelled PNA human centromere, red) were found in HLSC-injected mice livers (FITC-labelled mouse pan-centromeric chromosome, green). Mouse liver cells and HLSC were used as positive control for mouse- and human-specific hybridisation signals, respectively. Nuclei were stained with DAPI.

**Table S1: Primers used for PCR and qRT-PCR in this study**.

**Table S2: Mice used in the survival study**

**Movie S1: PBS-injected NSG/Ugt1^-/-^ mice show severe motor deficits compared to HLSC-injected NSG/Ugt1^-/-^ littermates.**

**Movie S2: HLSC-injected NSG/Ugt1^-/-^ mice at 21 days were comparable to the wt littermates.**
