# Supplementary material for: Human liver stem cells express UGT1A1 and improve phenotype of immunocompromised Crigler Najjar syndrome type I mice
Source: Sci Rep. 2020 Jan 21;10:887. doi: 10.1038/s41598-020-57820-2 (PMC6972964; doi:10.1038/s41598-020-57820-2)
Supplement: Supplementary file 1 — Supplementary Information. [file 41598_2020_57820_MOESM1_ESM.zip › Supplementary Information/Supplementary Methods.docx]

**Human liver stem cells express UGT1A1 and improve phenotype of immunocompromised Crigler Najjar syndrome type I mice**

Elvira Smeralda Famulari ^1$^, Victor Navarro-Tableros ^2$^, Maria Beatriz Herrera Sanchez ^2^, Giulia Bortolussi ^3^, Marta Gai^1^, Laura Conti^1^, Lorenzo Silengo^1,6^, Emanuela Tolosano ^1^, Ciro Tetta ^4^ , Andrés Fernando Muro ^3^, Giovanni Camussi ^5^, Sharmila Fagoonee ^6*^ and Fiorella Altruda ^1,6*^

**Characterisation of NSG/Ugt1^-/-^** **mice phenotype**

The resulting Ugt1^+/-^ mice from each generation were crossed with NSG wt mice for 7 generations. NSG genotype and depletion of B and T lymphocytes as well as natural killer cells were verified by PCR and flow cytometry, respectively (not shown). Thereafter, NSG/Ugt1^-/-^ mice were obtained by breeding NSG/Ugt1^+/-^ mice. Genotype was confirmed by PCR analysis of tail DNA and Western blot of liver proteins (Figure S6A and B).

**DiD staining of HLSC and in vivo analysis**

# HLSC were stained with the far-red fluorescent, lipophilic dye, DiD (DiIC_18_(5) solid (1,1'-Dioctadecyl-3,3,3',3'-Tetramethylindodicarbocyanine, 4-Chlorobenzenesulfonate Salt, ThermoFisher) according to the manufacturer’s instructions prior to injection in mice livers. On Day 0 and Day 11, images were acquired with IVIS 200 small animal imaging system (PerkinElmer, Waltham, MA, USA) using an excitation filter at 640 nm and an emission filter at 700 nm.

**Flow cytometry analysis**

Depletion of B and T lymphocytes and natural killer (NK) cells in NSG/Ugt1^-/-^ mice generated by the backcrosses were verified using FITC anti-mouse CD3ε (T lymphocytes, clone 145-2C11, BioLegend), PerCP/Cy5,5 anti-mouse/human CD45/B220 (B-lymphocytes, clone RA3-6B2, BioLegend) and APC anti-mouse CD335 (NKp46) (NK cells, clone.29A1.4, BioLegend) by flow cytometry (Beckton Dickinson). Characterization of different lines of HLSC (HLSC-6b used in the present study, as well as HLSC-2 and HLSC-16) was performed by cytofluorometric analysis, and anti-CD105, -CD29 and -CD73, phycoerythrin (PE) or fluorescein isothiocyanate (FITC)-conjugated, were used (CD105-FITC human, 130-098-774; CD29-FITC, human,130-101-256; CD73-PE, human, 130-095-182; Mouse IgG1-FITC,130- 113-761; Mouse IgG1-PE, 130-113-200; from Miltenyi Biotec). Anti-albumin used was from LSBio, Lifespan Biosciences (Albumin-FITC, LS-c68850; Isotype control FITC, LS-c149360). Cells were stained with antibodies and data were analysed using the CellQuest software (BD FACSCalibur).

For HNF4α staining, cells were isolated from PBS- or DiD+ HLSC-injected mice livers. Cells were fixed and permeabilised using the BD cytofix/cytoperm kit (554715, BDBioscience), stained with anti-HNF4α antibody and incubated with swine anti-rabbit FITC-conjugated secondary antibody (F0205, Agilent), acquired on a BD FACSVerse and analysed with FlowJO v10.

**Immunohistochemistry for E-cadherin and HNF4α**

HLSC and HaCAT cells grown on glass slides and sections of bioscaffolds containing HLSC were stained with anti-E-cadherin antibody (in-house) and anti-mouse biotinylated antibody and the ABC complex (Vector Labs) followed by exposure to 3, 3’-Diaminobenzidine (DAB, Roche).Nuclei were counterstained with hematoxylin.

**FISH analysis**

Mouse livers were perfused with liver perfusion medium and liver digest medium (ThermoFisher) at 37°C. Liver cells were isolated and centrifuged 1000 rpm 5 minutes and lysed in trisodium citrate dihydrate 5mg/ml, KCl 5.59 mg/ml 10 minutes at 37°C. Reaction was blocked with 3 drops of fixing solution (methanol: acetic acid 3:1). After centrifugation (1000 rpm 10 minutes), cells were incubated in fixing solution 15minutes RT. Then they were washed two times in fixing solution, resuspended in 30ul of fixing solution and dropped off on the slides. Chromosomes were denatured on slide in 70% formamide, 2X SSC for 2 minutes at 72°C. Then slides were dehydrated through a series of alcohol washes (70%, 95%, 100% ice cold ethanol). After drying at RT, Cy3-labelled PNA human centromere (Panagene, Daejeon, South Korea) and FITC-labelled mouse pan-centromeric chromosome (Cambio StarFISH, Cambridge, UK) probes were added simultaneously, the slides covered with coverslips and sealed with rubber cement. Hybridization was carried out for 16h a 37°C. The rubber cement and the coverslips were removed, and the slides washed using 2X SSC for 5 minutes at 37°C, formamide 50% in 2X SSC for 5 minutes at 37°C two times and finally 2X SSC for 5 minutes at RT two times. Nuclei were counterstained using 49,69-diamidino-2-phenylindole (DAPI) at a concentration of 100 ng/ml. Signals were visualized with fluorescence microscope (Nikon Eclipse 80i with ViCo).
