# Supplementary figures and images for: Human liver stem cells express UGT1A1 and improve phenotype of immunocompromised Crigler Najjar syndrome type I mice
Source: Sci Rep. 2020 Jan 21;10:887. doi: 10.1038/s41598-020-57820-2 (PMC6972964; doi:10.1038/s41598-020-57820-2)

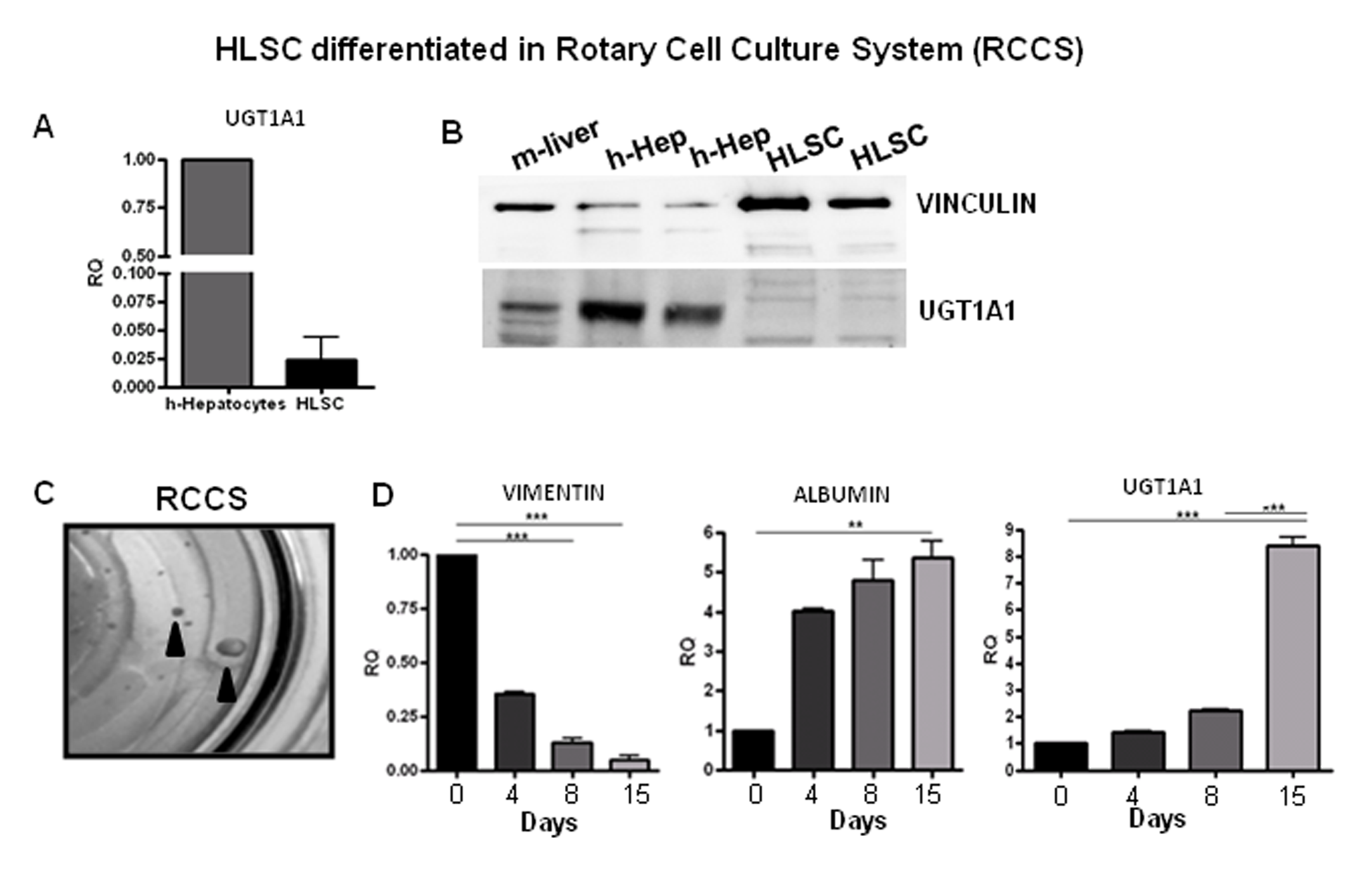

Supplement: Supplementary file 1 — Supplementary Information. [file 41598_2020_57820_MOESM1_ESM.zip › Supplementary Information/Figure S1.tif]

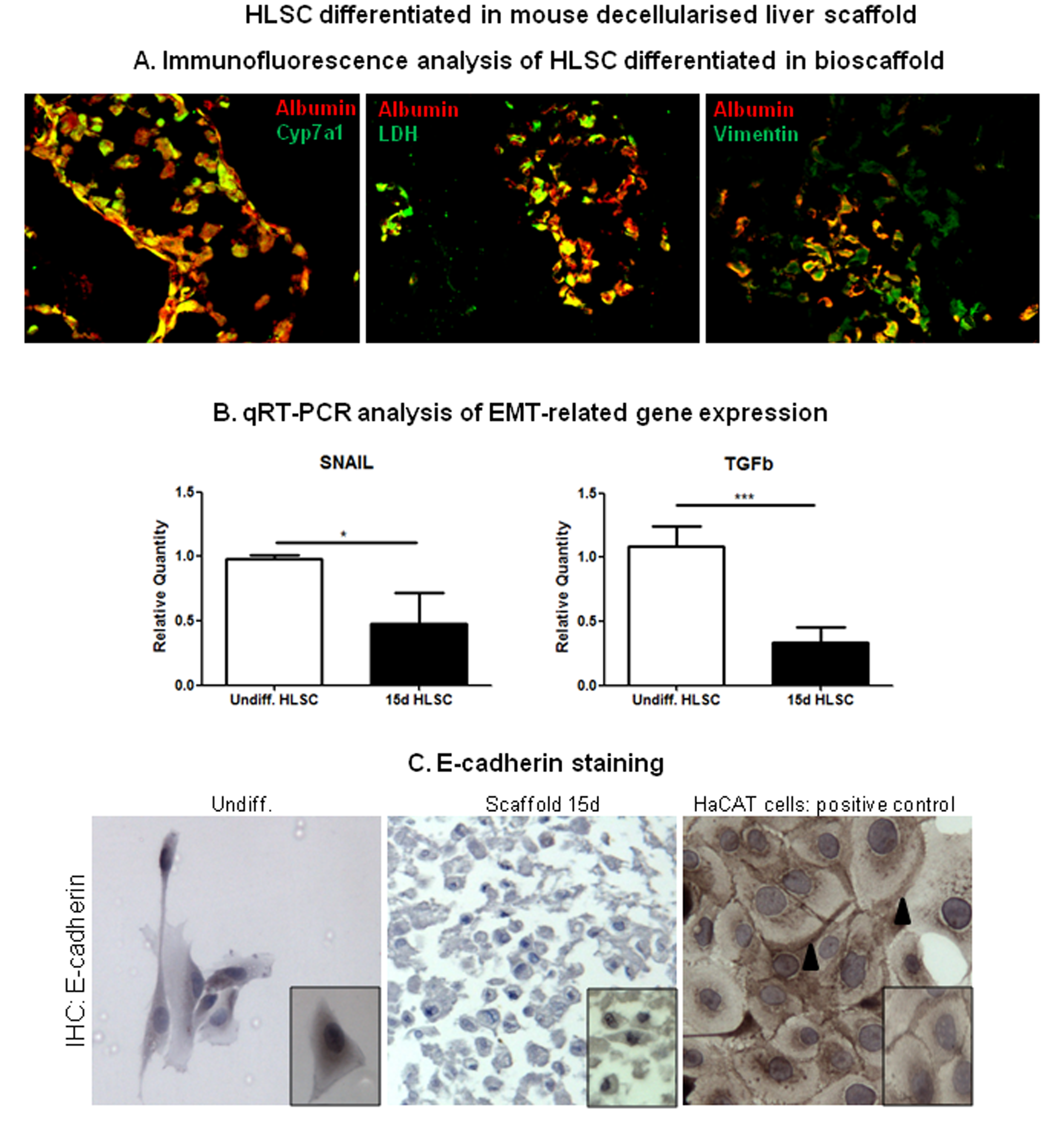

Supplement: Supplementary file 1 — Supplementary Information. [file 41598_2020_57820_MOESM1_ESM.zip › Supplementary Information/Figure S2.tif]

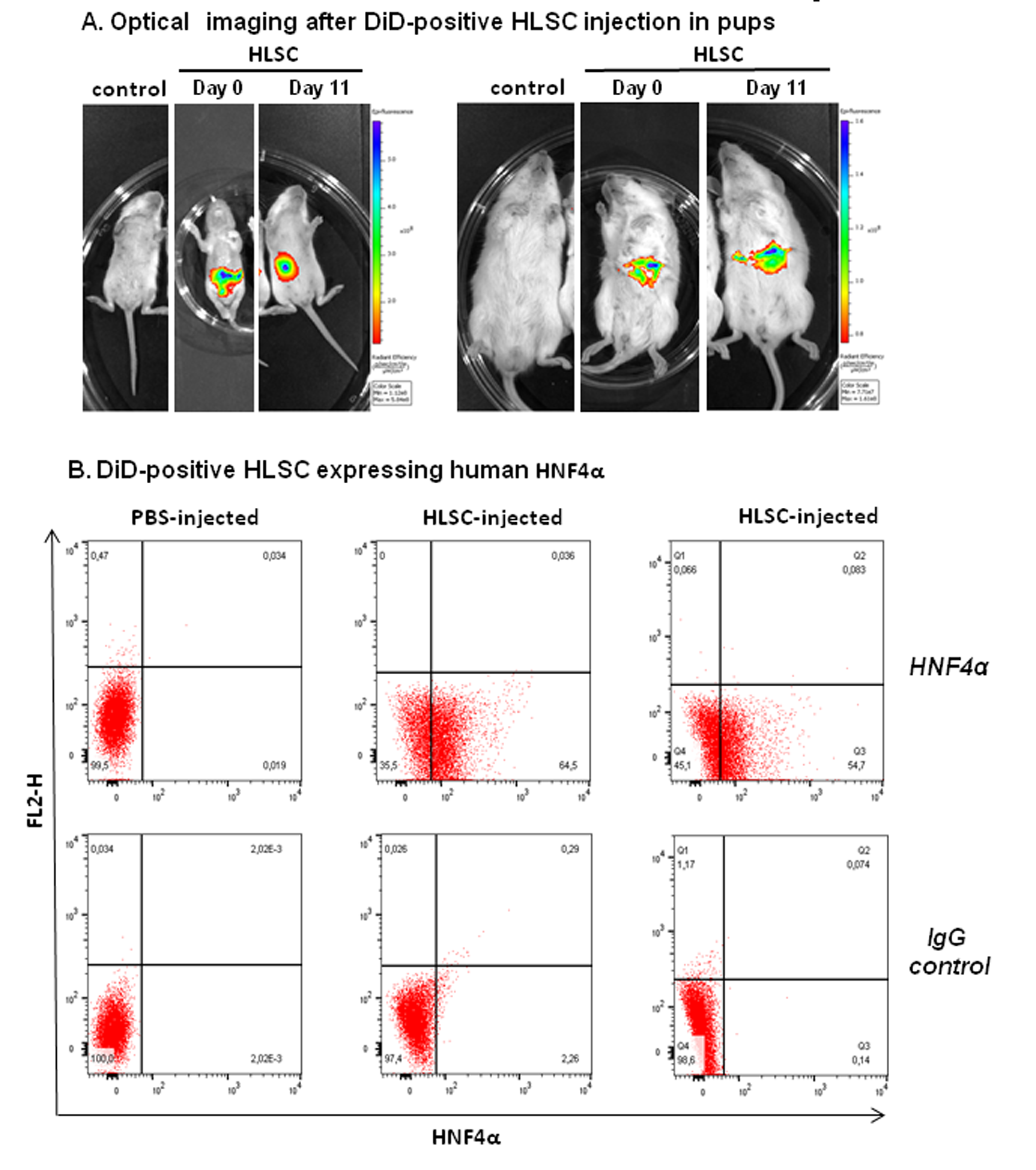

Supplement: Supplementary file 1 — Supplementary Information. [file 41598_2020_57820_MOESM1_ESM.zip › Supplementary Information/Figure S3.tif]

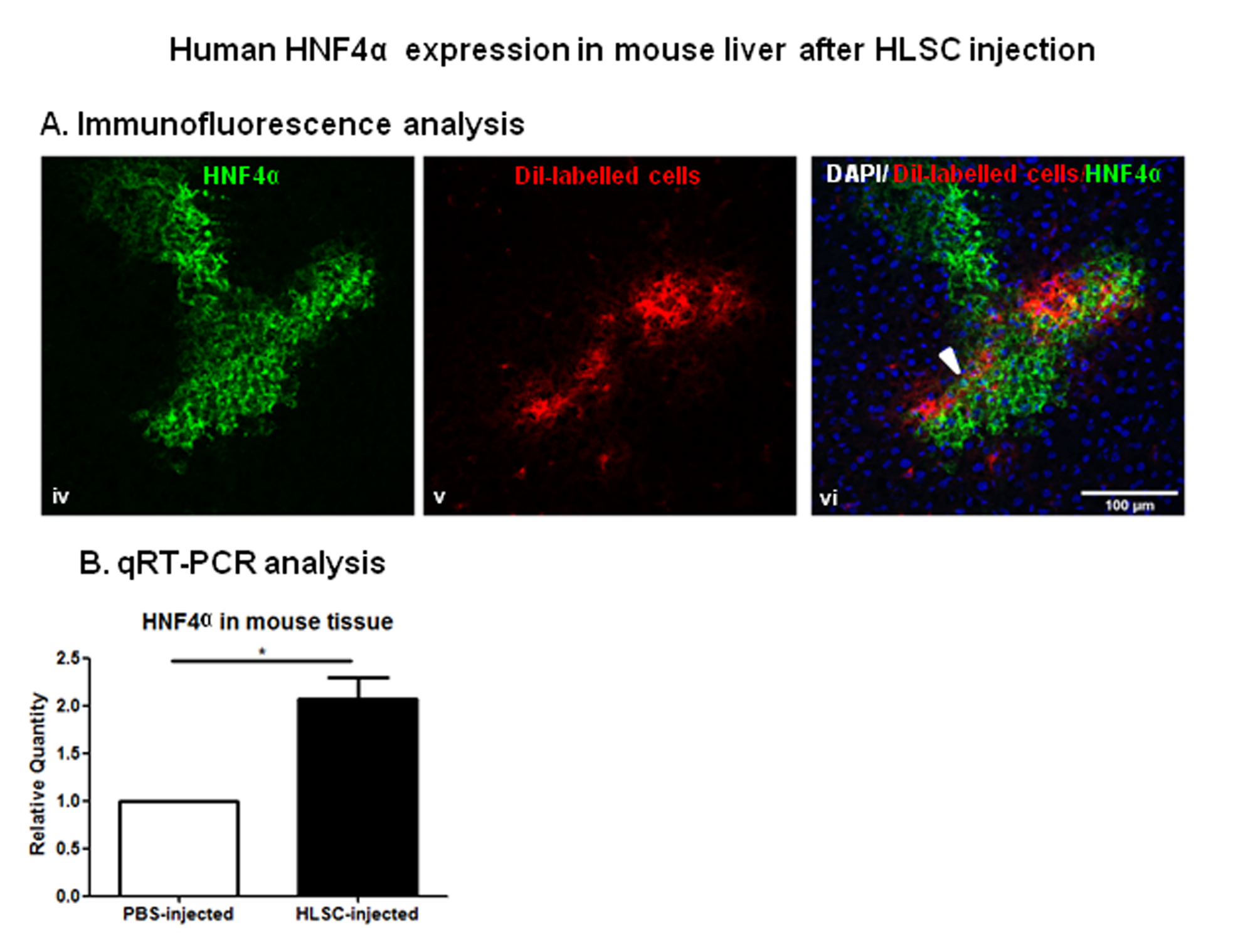

Supplement: Supplementary file 1 — Supplementary Information. [file 41598_2020_57820_MOESM1_ESM.zip › Supplementary Information/Figure S4.tif]

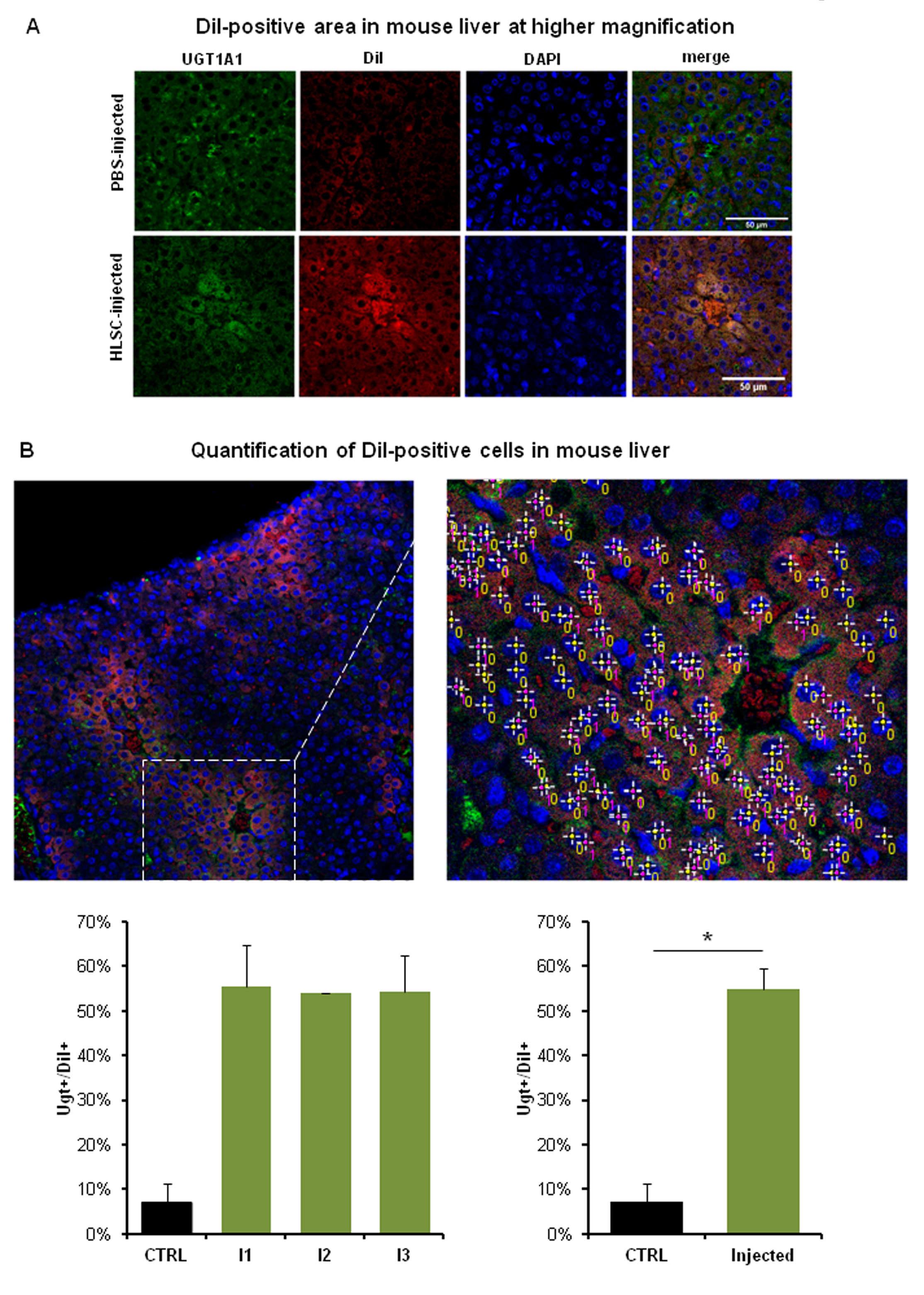

Supplement: Supplementary file 1 — Supplementary Information. [file 41598_2020_57820_MOESM1_ESM.zip › Supplementary Information/Figure S5.tif]

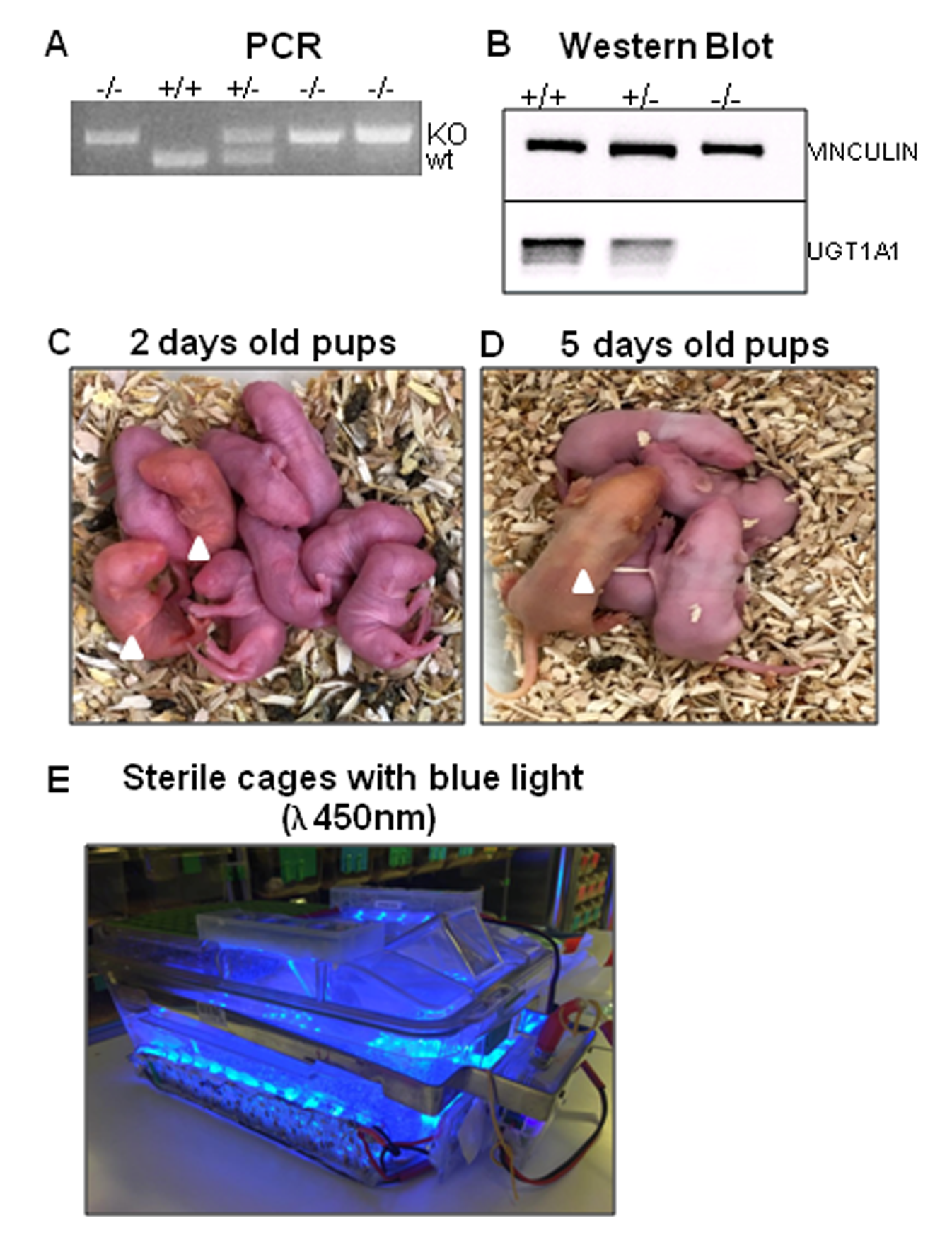

Supplement: Supplementary file 1 — Supplementary Information. [file 41598_2020_57820_MOESM1_ESM.zip › Supplementary Information/Figure S6.tif]

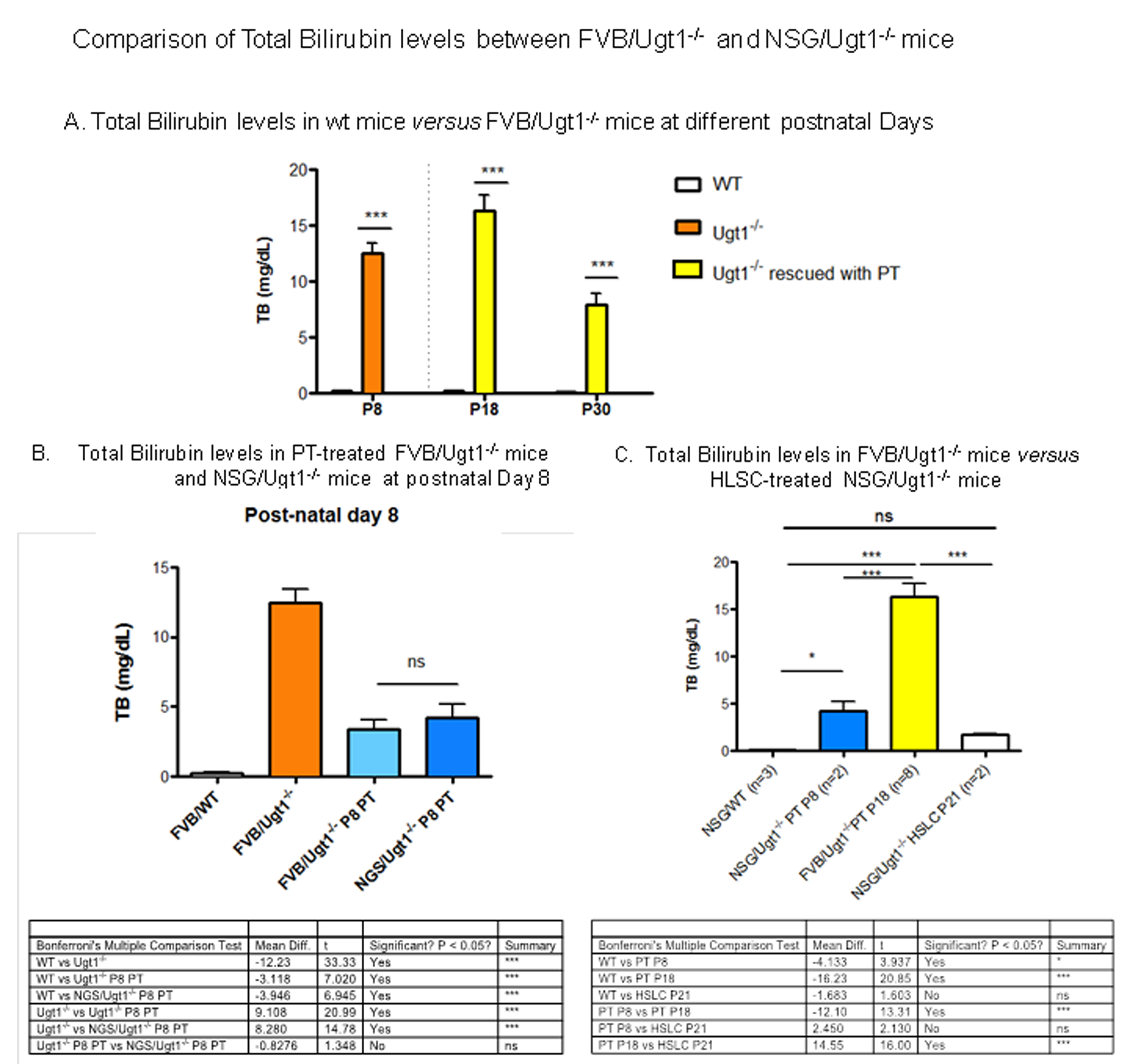

Supplement: Supplementary file 1 — Supplementary Information. [file 41598_2020_57820_MOESM1_ESM.zip › Supplementary Information/Figure S7.tif]

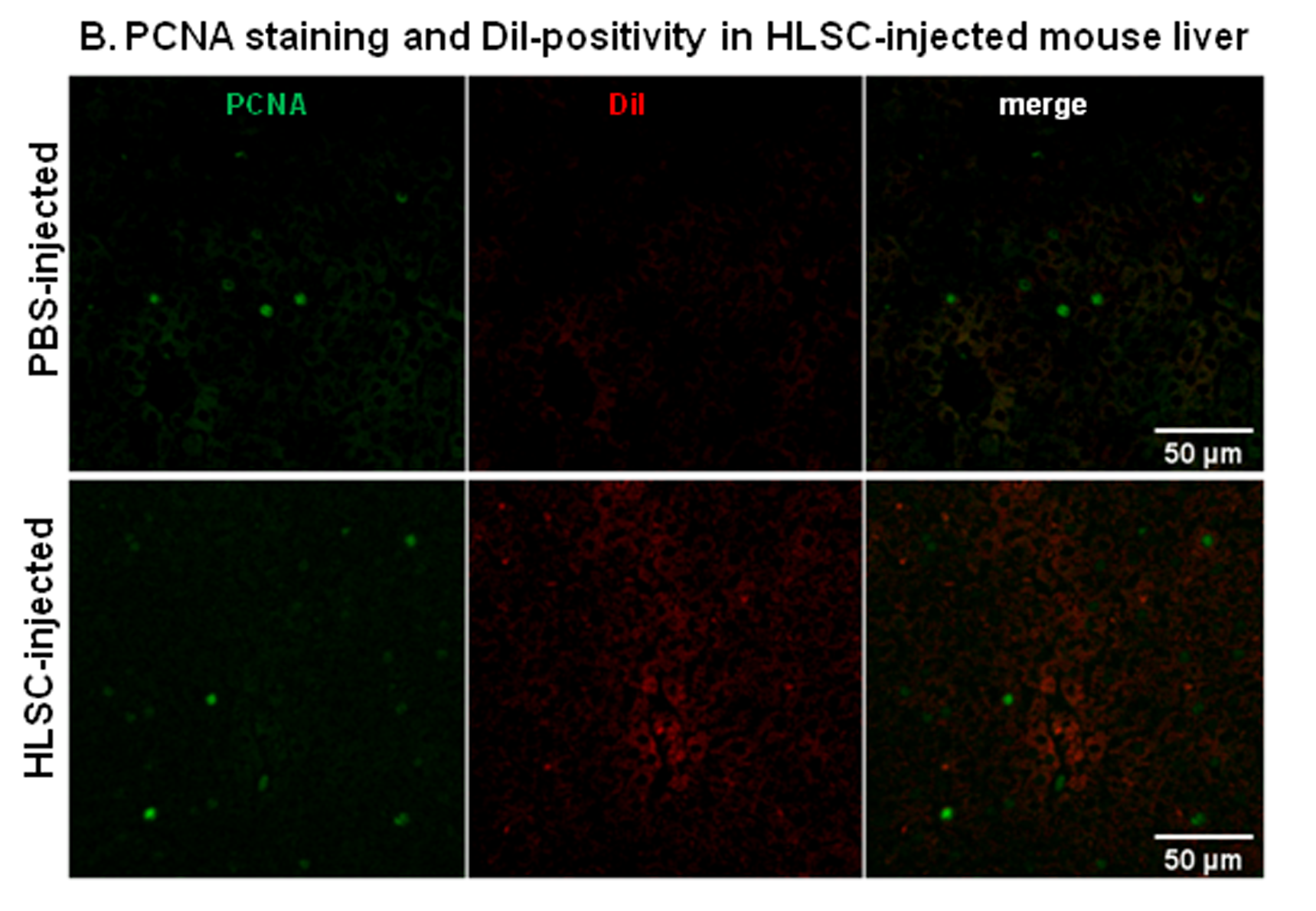

Supplement: Supplementary file 1 — Supplementary Information. [file 41598_2020_57820_MOESM1_ESM.zip › Supplementary Information/Figure S8.tif]

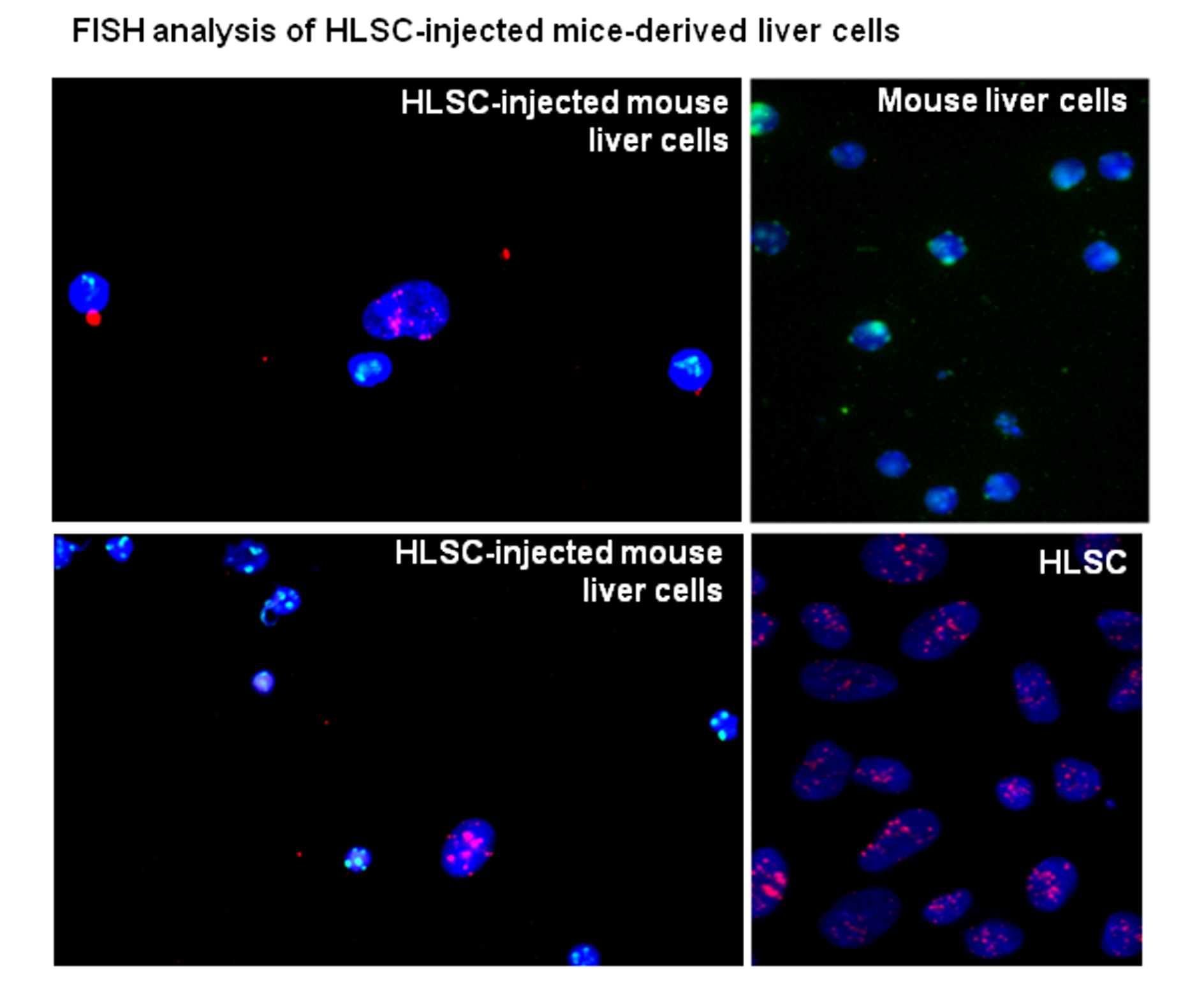

Supplement: Supplementary file 1 — Supplementary Information. [file 41598_2020_57820_MOESM1_ESM.zip › Supplementary Information/Figure S9.tif]
